# Longitudinal assessment of the bovine ocular bacterial community dynamics in calves

**DOI:** 10.1186/s42523-021-00079-3

**Published:** 2021-01-30

**Authors:** Alison C. Bartenslager, Nirosh D. Althuge, John Dustin Loy, Matthew M. Hille, Matthew L. Spangler, Samodha C. Fernando

**Affiliations:** 1 Department of Animal Science, University of Nebraska-Lincoln, Lincoln, NE USA; 2 Department of Food Science and Technology, University of Nebraska-Lincoln, Lincoln, NE USA; 3 School of Veterinary Medicine and Biomedical Sciences, University of Nebraska-Lincoln, Lincoln, NE USA; 4 School of Biological Sciences, University of Nebraska-Lincoln, Lincoln, NE USA

**Keywords:** Infectious bovine keratoconjunctivitis, 16S rRNA sequencing, Microbiota, *Moraxella*, *Mycoplasma*

## Abstract

**Background:**

Infectious Bovine Keratoconjunctivitis (IBK), commonly known as pinkeye, is one of the most significant diseases of beef cattle. As such, IBK costs the US beef industry at least 150 million annually. However, strategies to prevent IBK are limited, with most cases resulting in treatment with antibiotics once the disease has developed. Longitudinal studies evaluating establishment of the ocular microbiota may identify critical risk periods for IBK outbreaks or changes in the microbiota that may predispose animals to IBK.

**Results:**

In an attempt to characterize the establishment and colonization patterns of the bovine ocular microbiota, we conducted a longitudinal study consisting of 227 calves and evaluated the microbiota composition over time using amplicon sequence variants (ASVs) based on 16S rRNA sequencing data and culture-based approaches. Beef calves on trial consisted of both male (intact) and females. Breeds were composed of purebred Angus and composites with varying percentages of Simmental, Angus, and Red Angus breeds. Average age at the start of the trial was 65 days ±15.02 and all calves remained nursing on their dam until weaning (day 139 of the study). The trial consisted of 139 days with four sampling time points on day 0, 21, 41, and 139. The experimental population received three different vaccination treatments (autogenous, commercial (both inactivated bacteria), and adjuvant placebo), to assess the effectiveness of different vaccines for IBK prevention. A significant change in bacterial community composition was observed across time periods sampled compared to the baseline (*p* < 0.001). However, no treatment effect of vaccine was detected within the ocular bacterial community. The bacterial community composition with the greatest time span between sampling time periods (98d span) was most similar to the baseline sample collected, suggesting re-establishment of the ocular microbiota to baseline levels over time after perturbation. The effect of IgA levels on the microbial community was investigated in a subset of cattle within the study. However, no significant effect of IgA was observed. Significant changes in the ocular microbiota were identified when comparing communities pre- and post-clinical signs of IBK. Additionally, dynamic changes in opportunistic pathogens *Moraxella* spp*.* were observed and confirmed using culture based methods.

**Conclusions:**

Our results indicate that the bovine ocular microbiota is well represented by opportunistic pathogens such as *Moraxella* and *Mycoplasma.* Furthermore, this study characterizes the diversity of the ocular microbiota in calves and demonstrates the plasticity of the ocular microbiota to change. Additionally, we demonstrate the ocular microbiome in calves is similar between the eyes and the perturbation of one eye results in similar changes in the other eye. We also demonstrate the bovine ocular microbiota is slow to recover post perturbation and as a result provide opportunistic pathogens a chance to establish within the eye leading to IBK and other diseases. Characterizing the dynamic nature of the ocular microbiota provides novel opportunities to develop potential probiotic intervention to reduce IBK outbreaks in cattle.

**Supplementary Information:**

The online version contains supplementary material available at 10.1186/s42523-021-00079-3.

## Introduction

One of the most significant diseases in beef cattle is Infectious Bovine Keratoconjunctivitis (IBK), commonly known as pinkeye [1–3]. IBK costs the US beef cattle industry more than 150 million US dollars annually and a recent survey ranked it as the third highest animal health challenge with 12.5% of producers indicating IBK was the most significant animal health challenge [4]. The clinical signs of IBK are variable with afflicted animals exhibiting corneal ulceration and edema [5, 6]. Additionally, this disease causes ocular pain and varying degrees of corneal scarring, corneal rupture and permanent blindness depending on the severity of the infection [5, 6].

Although many factors contribute to an IBK infection, *Moraxella bovis* is typically the principal etiologic agent of IBK [7]. Other agents such as *Moraxella bovoculi* and *Mycoplasma bovoculi* have also been associated with IBK outbreaks and their presence may predispose the animal to disease [1, 8, 9]. It is suggested that *Moraxella* and *Mycoplasma* enter the eye via face flies or as an effect of secretion after irritation of the eye due to cuts from tall grass, feed, or UV light damage from the sun [10]. These pathogens can also be carried by asymptomatic animals in the eye and nasopharynx, potentially serving as a reservoir for additional infections [11, 12].

Currently, vaccination for IBK is limited to the use of commercial vaccines that have been fully licensed by the USDA or autogenous vaccines (a vaccine prepared from killed cultures of organisms isolated from the same herd) [1, 6, 13, 14], which have shown variable efficacy [15]. One potential reason for the lack of effective prevention strategies for IBK is a limited understanding of the ocular microbiota in cattle. Specifically, there is a knowledge gap in how the ocular microbiota is established or how it contributes to the development of IBK. Changes in the ocular microbiota that may lead to increased opportunistic pathogen colonization are poorly understood due to the lack of studies investigating the bovine ocular microbiota [16]. Within the human ocular microbiota, it is suggested that immunoglobulin and neutrophil response affect the ocular microbiota [17]. In a recent study, Cullen et al. [2] utilized high-throughput sequencing of the 16S rRNA gene to compare ocular bacterial communities of calves who developed IBK against those calves who did not show IBK symptoms. This first study of the bovine ocular microbiota demonstrated no large-scale community differences between cattle infected with IBK and cattle without IBK. However, they reported Faith’s Phylogenetic Diversity [18] was greater in cattle infected with IBK than the control cohort. Additionally, Cullen et al. [2], concluded that there was no variation in *Moraxella* spp. abundance among the two cohorts even though *Moraxella* was identified as the most abundant genus. With many studies describing the dynamic nature of the microbiota [19], it is critical to monitor microbiota changes over time to identify the stable phenotype of the ocular microbiota and how the ocular microbiota changes in response to perturbation. We hypothesize that a longitudinal analysis of the bovine ocular microbiome will provide new information relative to the adaptability of the ocular microbiome to change and help identify potential windows of time when a calf is more susceptible to IBK. As such, this study characterizes the establishment and dynamic changes in the ocular microbiota in healthy calves and calves that developed IBK to identify windows of opportunity that may increase disease susceptibility. Therefore, in this study, to better assess the bacterial species composition within the bovine ocular microbiota, we performed a longitudinal assessment of the bacterial species composition over 139 days that included four different sampling time points to evaluate the bacterial community changes using 16S rRNA sequencing.

## Results

In an attempt to characterize the establishment and colonization patterns of the bovine ocular bacterial community until weaning, we conducted a longitudinal study consisting of 227 calves and evaluated the microbiota composition over time using amplicon sequence variants (ASVs) based on 16S rRNA sequencing data. Beef calves on trial consisted of both males (intact) and females. Breeds were composed of purebred Angus, and red and black composites with varying percentages of Simmental, Angus, and Red Angus breeds. Average age at the start of the trial was 65 days ±15.02 and all calves remained nursing on their dam until weaning (day 139 of the study). The trial consisted of 139 days with four sampling time points of day 0, 21, 41, and 139 days. The experimental population received three different vaccination treatments (autogenous, commercial (both inactivated bacteria), and adjuvant placebo), to assess the effectiveness of different vaccines for IBK prevention. However, no significant change in the bacterial community composition was detected based on vaccine treatment used and therefore the subsequent analysis was performed to evaluate bacterial community changes in the ocular microbiome over time and factors that may lead to IBK infection.

Additionally, an experimental population consisting of 30 heifers was utilized to evaluate the effect of perturbation of one eye on the bacterial community composition of the other eye. The experimental design included, swabbing of both the left and right eye in 10 animals on day 0 (cohort A), swabbing of the left eye in 10 animals on day 0 (cohort B), and swabbing of right eye in 10 animals on day 0 (cohort C), followed by swabbing of left and right eye in all animals for a second time 17 days later (*n* = 30). All swabs collected were processed and the 16S rRNA based bacterial community was analyzed in a manner similar to the longitudinal study.

### Effect of perturbation of one eye on the microbial community of the other eye

**Fig. 1:**
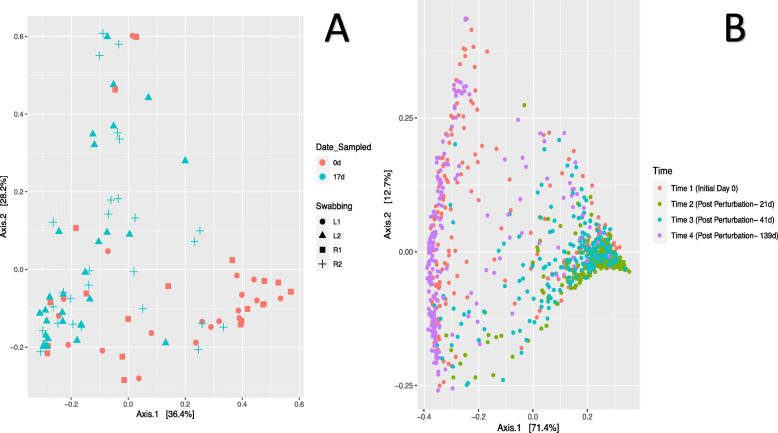
Principal Coordinate analysis (PCoA) demonstrating between sample variations in beta-diversity. PCoA plot was generated using a weighted UniFrac distance matrix. **a** The PERMANOVA analysis (*p* < 0.001) and PCoA demonstrated, irrespective of which eye was swabbed the resulting perturbation to affect the other eye. As such, no significant difference in bacterial community composition was detected in respect to eye swabbed. This is apparent in the PCoA plot with no clustering. Color scheme; Red – day 0 and Teal – day 17; Shape scheme; Circle – left eye swabbed on day 0, Triangle – left eye swabbed on day 17, Square – right eye swabbed on day 0 and Cross – right eye swabbed on day 17. **b** PERMANOVA analysis demonstrated microbial communities to be significantly different based on time of sampling (*p* < 0.05). This  is apparent in the clustering pattern. Time points 1 and 4 demonstrated similar community structure that was different from time points 2 and 3. Microbial community composition and abundance in time points 2 and 3 were similar to each other. The major contributors of microbial community composition included, time of sampling, age, and ulcer positive. Color scheme; Red – time point 1, Green - time point 2, Teal – time point 3, and Purple – time point 4. All four time points are associated with a perturbation due to the swabbing

An experiment was performed on a different cohort, but reared in the same environment and genetic background, consisting of bovine heifers to investigate if the perturbation of one eye would lead to changes in the microbial community in the other eye. Following quality filtering, the resulting samples contained 2,462,425 reads with an average of 24,624 reads per sample. These reads were binned into 1434 ASVs. A weighted UniFrac distance matrix [20] was used to visualize bacterial community composition changes using Principal Coordinate Analysis (PCoA) (Fig. 1a). No clustering was observed based on which eye was sampled. However, bacterial community clustering was observed based on time of sampling. PERMANOVA analysis further supported this observation where the only significant change in the bacterial community composition was based on time (*p* < 0.001) with eye sampled and cohort having *p*-values of *p* = 0.678, and *p* = 0.404 respectively. To further evaluate the effect of eye sampled on subsequent community changes, we independently investigated each cohort at day 17 to evaluate if swabbing the left or right eye 17 days earlier resulted in bacterial communities to be different in the right and left eye. Weighted UniFrac distance [20] based PCoA (Figure S2) and PERMANOVA analysis further supported our previous findings as no significant difference was identified between left and right eye (Cohort A: *p* = 0.37, Cohort B: *p* = 0.25, and Cohort C: *p* = 0.659). Additionally, Cohort A (both eyes swabbed at day 0 and 17), revealed there was no significant difference between left and right eye. However, sampling time displayed significantly different bacterial community composition suggesting the swabbing of the eye is a perturbation event.

### Changes in the bovine ocular microbiota over time

**Fig. 2:**
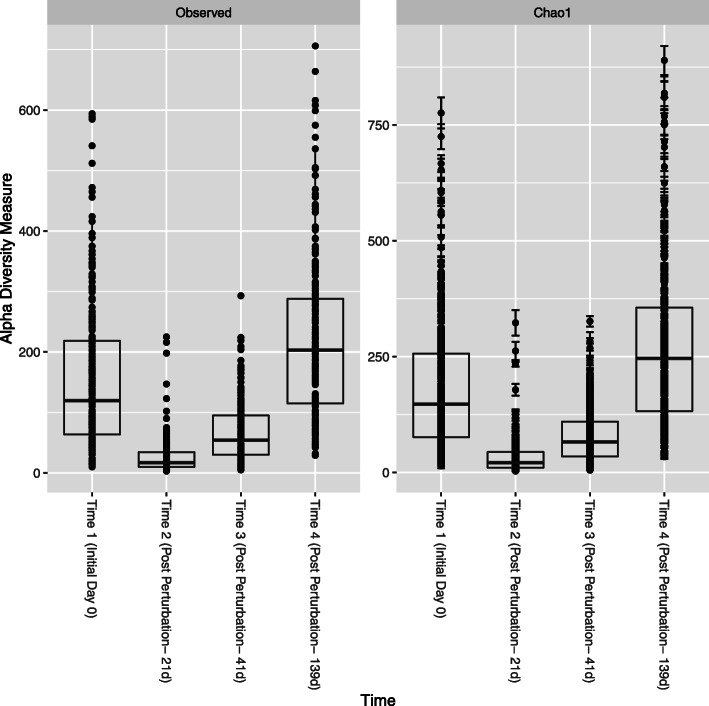
Changes in alpha diversity in time periods sampled of all samples (*n* = 227). Observed ASVs (**a**) and Chao1 estimates (**b**). Significant differences in alpha-diversity were identified between the sampling periods where the greatest diversity was found in time period 4. After initial swabbing for baseline community, the diversity significantly decreased and recovered over time in subsequent samplings. The initial swabbing, would be deemed as the first perturbation or disruption to the ocular microbiota

Following quality filtering, the resulting data contained 862 samples with 23,417,226 reads with an average of 27,166 reads per sample. These reads were binned into 5820 amplicon sequence variants (ASVs). To ensure read depth did not influence community changes identified, reads were rarefied to an even depth for alpha diversity measures (3022 reads). Changes in bacterial community diversity were evaluated using alpha diversity metrics for the entire cohort of animals (*n* = 227). Measures used included observed ASVs and Chao1 estimates [21]. Alpha diversity using both observed ASVs and Chao1 estimates displayed significant differences in bacterial diversity between all-time points demonstrating dynamic changes that occur in the bovine ocular microbiota. The least diversity was identified 21 days after initial swabbing where bacterial diversity was the lowest, after which, the diversity increased to near-baseline levels (day 0) in subsequent samplings (Fig. 2).

To further evaluate global changes in the ocular bacterial community over time, a principal coordinate analysis was performed on the normalized dataset using a weighted UniFrac distance matrix [20]. PERMANOVA results revealed significant differences in bacterial community composition based on time sampled, age at sampling, hide color, sex, and breed (*p* < 0.001, *p* = 0.034, *p* = 0.006, *p* = 0.010, and *p* = 0.024 respectively). However, vaccine treatment and presence or absence of IBK infection did not show a significant effect on the microbial community (*p* > 0.101, and *p* > 0.848, respectively) (Fig. 1b). Interestingly, days 0 & 139 and days 21 & 41 displayed similar bacterial community structure. However, community structure of days 21 and 41 were significantly different from days 0 and 139. This suggests the bacterial community may be changing after each ocular swabbing as the swabbing of the eye can be considered as a perturbation. However, the longer recovery period between days 41 and 139 suggests that the ocular microbiome is able to revert back and reach a community similar to baseline levels over time. To further evaluate this observation, we performed a hierarchical clustering of the bacterial communities. This analysis confirmed our previous observation where bacterial communities clustered based on sampling time with days 0 & 139 and days 21 & 41 clustering together.

### Core measurable microbiota in the bovine ocular microbiota

**Fig. 3:**
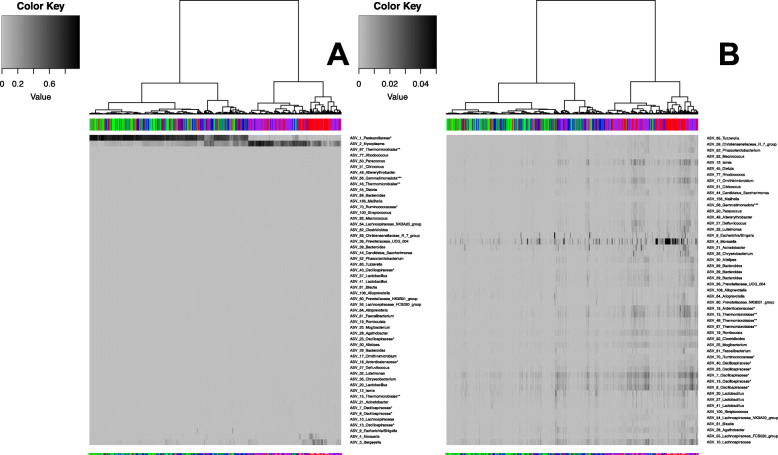
Heat-map showing the distribution of “Core” ASVs representing each time period. Hierarchical clustering of ASV abundance based on sampling time period clearly shows clustering based on sampling period. Red and purple represent sampling periods 1 and 4 and green and blue represent time periods 2 and 3. Samples were individually clustered using hierarchical clustering based on time period. The top 54 most abundant ASVs (5A) and the remaining top 51 ASVs (5B) are shown. Figures **a** and **b** are drawn using different scales due to differences in abundance. The top 3 ASVs include ASV_1_Pasteurellaceae, ASV_2_Mycoplasma, and ASV_3_Weeksellaea. (Key- * denotes taxa at family level, ** denotes taxa at order level, and *** denotes taxa at class level)

It is well documented that the microbiota varies between individuals [19]. As such, to look beyond variation in the microbiota and to identify the core bacterial community present within the ocular microbiota, we identified the core measurable microbiota (CMM) by identifying ASVs that were present in at least 80% of total samples collected. This analysis only identified three ASVs to be part of the CMM and accounted for 80% of total reads in the dataset. The ASVs identified belonged to families *Pasteurellaceae*, *Weeksellacea,* and genus *Mycoplasma*. The distribution of the core ASVs across time points are shown in Figure S3. To further evaluate the taxa that contributes to the differences in the community structure displayed in the Principal Coordinate Analysis, we identified core microbial ASVs present at each time point using a criterion of ASVs represented in 75% of the animals at each time sampled. For time period one, 29 ASVs were identified accounting for 38% of the total reads. Additionally, 3, 5, and 40 ASVs accounting for 79, 80, and 84% of the total reads for each time point were identified for time periods two, three, and four, respectively. Hierarchical clustering of these core ASVs based on abundance displayed clear clustering based on time point where days 21 and 41 clustered closely together whereas, days 0 and 139 clustered together away from days 21 and 41 (Fig. 3). Three major ASVs were observed in these clusters and included family *Pasteurellaceae,* and genera *Mycoplasma,* and *Moraxella.* Further analysis of the ASV belonging to family *Pasteurellaceae* displayed that the abundance of this ASV was negatively associated with the abundance of *Mycoplasma.* The ASV belonging to family *Pasteurellaceae* was higher in abundance in day 21 and 41 samplings and genera *Mycoplasma* and *Moraxella* were higher on abundance in days 0 and 139.

This analysis further confirmed the structuring of bacterial communities by time and also suggested that on days 21 and 41 the community is changing rapidly with the loss of diversity as the few core ASVs identified represented 79 and 80% of the total reads sequenced for those two time periods.

In addition to changes in the CMM across time periods, we also investigated the differential abundance of ASVs across sampling time points using total bacterial community composition. As such, we found 140 unique differential ASVs among all four time point comparisons. Among those ASVs we see patterns of taxa being higher in abundance during days 0 and 139 (Figure S4).

### Relationship between bacterial ASVs and IBK outbreak

**Fig. 4:**
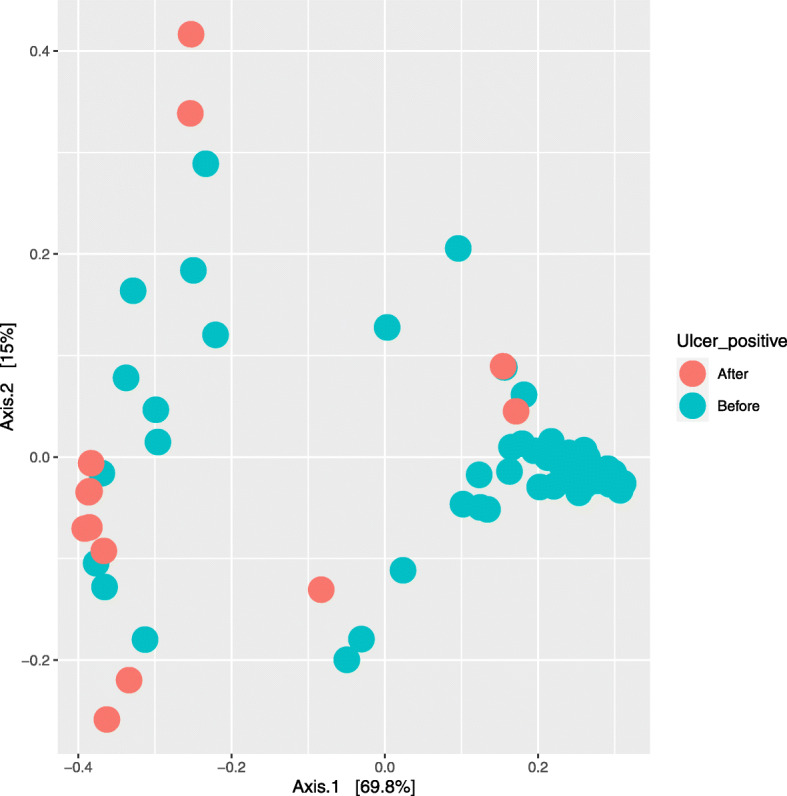
Principal Coordinate analysis (PCoA) demonstrating microbial community differences before and after ulcer formation. A significant difference in community composition was detected when samples before infection and after ulcer formation was analyzed. The PCoA was performed using a weighted UniFrac distance matrix. Color scheme; Teal –before ulcer and Red- after ulcer

**Fig. 5:**
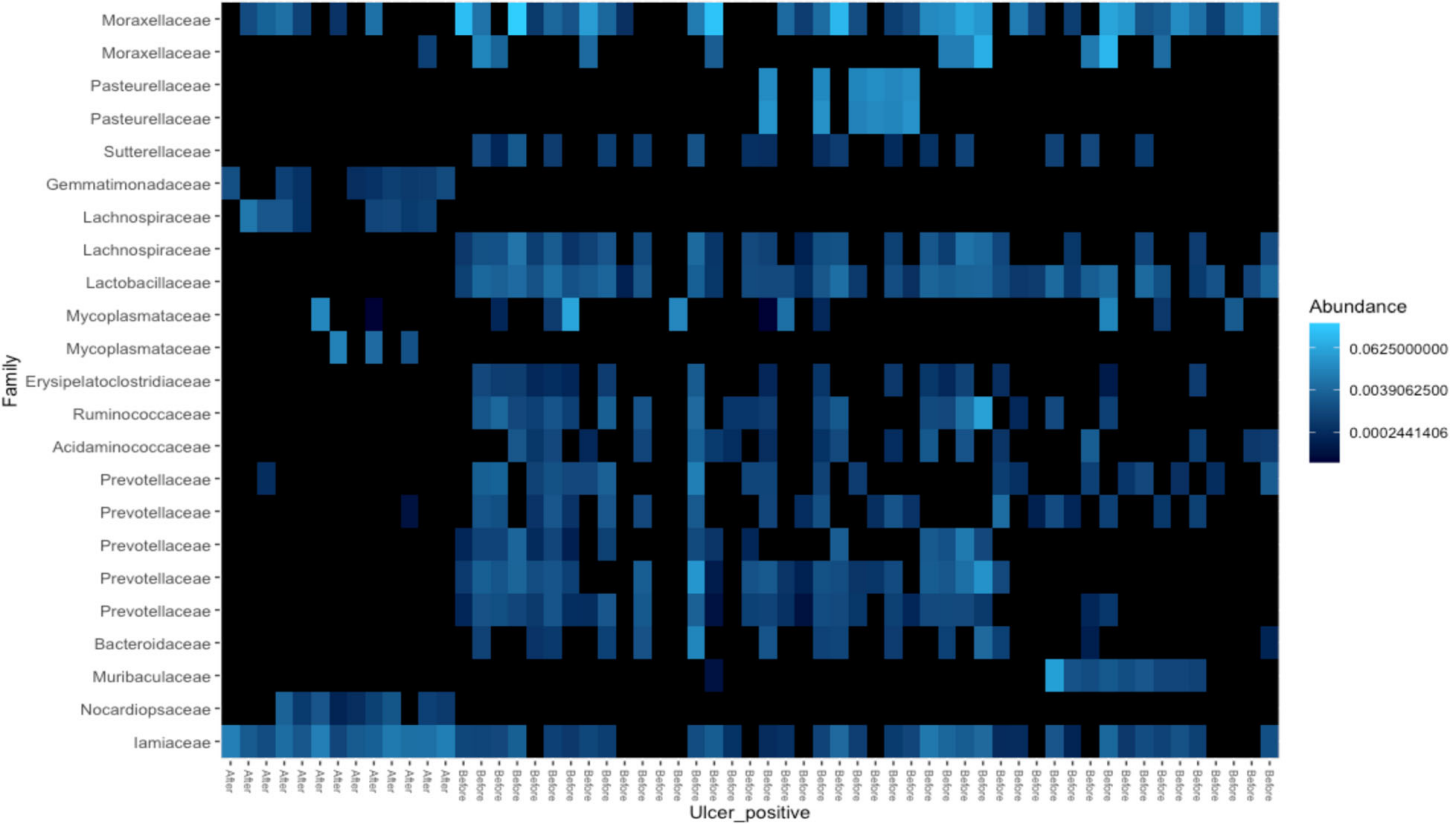
Heat-map showing differential ASVs identified within the bacterial community of ulcer positive animals before and after IBK infection. The major differences in community structure was driven by ASVs belonging to families *Lachnospiraceae* and *Prevotellaceae*

During the study period, 19 animals out of 227 were identified with IBK and were treated and given an ulcer score (Table S1). The IBK infections were observed from mid-June to the end of September (days 21–118 of the study). We evaluated the bacterial community composition of the animals treated for IBK using the sampling time points collected prior to detection of IBK infection and after IBK infection using a weighted UniFrac distance matrix. Clustering, based on the PCoA plot, clearly show differences between pre- and post- IBK infection (Fig. 4). PERMANOVA analysis did detect a significant change in samples taken pre and post IBK infection (*p* < 0.003) in the overall structure of the bacterial community suggesting a composition shift within the ocular microbiome. Additional investigation of the abundance of *Mycoplasma* and *Moraxella* pre- and post-IBK infection demonstrated *Mycoplasma* abundance to significantly increase (*p* < 0.001) in samples collected after IBK infection (from 0.77 ± 0.031% to 1.67 ± 0.021%) and *Moraxella* abundance to significantly decrease (*p* < 0.001) in samples collected after IBK infection (from 0.002 ± 0.0013% to 0.00014 ± 0.0002%). Further analysis of the differential ASVs identified before and after IBK infection belonged to the families such as *Lachnospiraceae* and *Prevotellaceae* with an increase in abundance post infection (Fig. 5). Interestingly, the only ASV identified from family *Mycoplasmataceae* and the single ASV identified from family *Moraxellaceae* was low in abundance in animals pre- IBK infection. However, post- IBK infection, abundance of the two ASVs increased. In addition, PERMANOVA analysis identified pre- and post- IBK infection bacterial community composition to be significant (*p* < 0.001).

### Abundance of known IBK predisposes (*Mycoplasma* spp. and *Moraxella* spp.) in the bovine ocular microbiota

Previous studies have reported *Mycoplasma* spp*.* and *Moraxella* spp. to be the major pathogens that predispose cattle to IBK [1, 8, 9]. The mean abundance of these taxa in the current dataset is shown in Figure S5. Although the relative abundance was low, *Moraxella* spp*.* were identified in all time points, with the highest abundance during the first time period suggesting that *Moraxella* spp. may be common inhabitants of the bovine ocular microbiota. However, *Mycoplasma* spp. abundance was much greater than the abundance of *Moraxella* spp. in time period four.

### Effect of immunoglobulin A (IgA) on structuring of the bovine ocular bacterial community

Previous studies of the human ocular microbiota have suggested that immunoglobulin and neutrophil levels within the eye affect the ocular microbiota [22]. To determine if IgA levels affect the bovine ocular microbiota, we evaluated IgA levels in animals with significantly different ocular bacterial community structure. IgA concentrations did not differ in animals with different bacterial community composition suggesting IgA concentration was not a driver of the ocular microbiota in this study (Figure S6).

### Prevalence of ocular *Moraxella* spp. over time

Culture results obtained at different time points starting from baseline sampling on day 0 through day 139 were used to identify if samples contained culturable *Moraxella* spp. This analysis displayed significant changes in the prevalence of *Moraxella* spp. over time, consistent with the changes in the relative abundance identified by 16S rRNA analysis over time (Figure S7). When total *Moraxella spp.* (*Moraxella bovis* and *Moraxella bovoculi*) were examined, a statistically significant increase in samples containing *Moraxella* spp. was observed on day 21, while a significant decrease in samples containing *Moraxella* spp. was observed on day 139 (Figure S7a).

### Functional differences in the ocular microbiome

In addition to assessing taxonomic differences in the bovine ocular microbiome over time, we predicted functional genes based on taxonomic information of the microbial community composition using Phylogenetic Investigation of Communities by Reconstruction of Unobserved States PICRUSt2 [23]. PICRUSt2 identified 7364 functional genes within our dataset. Alpha and beta diversity analysis displayed similar results to what was observed in taxonomy based analysis (Figures S8 and S9), where day 0 and 139 clustered together away from days 21 and 41. Days 21 and 41 displayed similar functional gene composition. Additionally, a PERMANOVA analysis was performed, using a Bray-Curtis distance matrix [24]. Results revealed time sampled, hide, sex and breed to all be significantly different (*p* < 0.001, *p* = 0.004, *p* = 0.005, and *p* = 0.028, respectively). Whereas vaccine treatment, age, and ulcer positive cattle had no significant affect (*p* < 0.178, *p* = 0.052, and *p* = 0.785, respectively). These results suggest that in the bovine ocular microbiome taxonomic changes are predictive of functional changes.

To further assess functional differences across time periods sampled, we identified differential KOs present between samplings. A total of 122 differential KOs were identified and is shown in Figure S10. Similar to the changes in ASV abundance, the differential KOs displayed clustering where time periods 1 & 4 and 2 & 3 had similar gene profiles.

## Discussion

IBK outbreaks have significant economic consequences to the cattle industry. With the lack of effective prevention methods to control IBK, the cattle industry is faced with the challenge of developing novel methods to prevent, control, and mitigate IBK outbreaks. Although outbreaks of IBK are unpredictable and vary from year to year, Midwestern beef herds in the United States have published estimates of 4.97 cases/100 animals [25]. The prevalence of IBK during this study was higher than these estimates at 7.9%, and averaged 18.5% (24.6, 7.9, 23.0%) over the last 3 years (data not shown), however prevalence is variable and unpredictable under field conditions. There has been an increasing emphasis on microbiota investigations to harness the therapeutic potential of the microbiota. As such, novel opportunities may be available to develop innovative methods to prevent, control, and monitor IBK outbreaks in cattle. In this study we investigated the bovine ocular microbiota over time to evaluate the bacterial compositional changes before and after subsequent perturbations (ocular swabbing(s)) to investigate the establishment and dynamic changes occurring in the bovine ocular microbiota. We phenotyped the microbiota using 16S rRNA based sequencing of the V4 hypervariable region, culture-based approaches to evaluate *Moraxella bovis* and *Moraxella bovoculi* prevalence, and recorded IBK infection occurrence.

### Perturbation of one eye leads to changes in the microbial community composition of the other eye

The PERMANOVA analysis (*p* < 0.001) and PCoA demonstrates that irrespective of which eye was swabbed, the resulting perturbation affected the other eye. As such, no significant difference in bacterial community composition was detected in respect to eye swabbed. Therefore, this suggests that a perturbation in the ocular microbiome of one eye is not just limited to that eye. As a result, similar changes occur in the other eye. This observation is not limited to this study, a study by Cavuoto et al. investigated the ocular surface microbiome in children and reported that the left and right eyes have similar bacterial community composition [26]. As such, we show that the ocular microbiome in calves is similar between the eyes and the perturbation of one eye results in similar changes to occur in the other eye and that swabbing is a significant perturbation event.

### The window for re-establishment of the ocular microbiota is long

The bovine ocular microbiota changed significantly over the four time periods sampled. The diversity of the microbiota dramatically decreased after the first swabbing and was lowest when sampled 21 days after the first swabbing. At 41 days post swabbing (20 days after the second swabbing) the bacterial diversity slightly increased relative to the second swabbing but remained significantly low compared to the initial swabbing. However, with time the bacterial diversity increased and re-established at diversity levels similar to initial baseline swabbing 139 days later (98 days after the 3rd swabbing) (Fig. 2). This demonstrates that when a change/perturbation occurs in the bovine ocular microbiota, time for recovery could be long and our findings suggest that this time interval can be between 21 and 98 days. During this time the ocular microbiome may be more vulnerable for opportunistic pathogens to colonize and cause disease. However, more frequent swabbing at different time intervals will provide information to help characterize the recovery time more accurately. The prolonged recovery time suggests that perturbations of the ocular microbiota due to environmental factors such as cuts from tall grass, feed, or UV light from the sun [10] could lead to changes in the ocular microbiota that provide a window of opportunity for opportunistic pathogens such as *Moraxella* spp*.* and *Mycoplasma* spp. to establish infections and potentially lead to IBK. Additionally, the immune system of young animals is rapidly developing, and subsequent maturation of the ocular immune system may affect microbial diversity [27, 28].

Further investigation of factors that influence the ocular bacterial community in cattle using PERMANOVA analysis revealed time period sampled, age at sampling, hide color, sex, and breed (*p* < 0.001, *p* = 0.034, *p* = 0.006, *p* = 0.010, and *p* = 0.024, respectively) to significantly affect the bacterial community. Previous studies have reported the human ocular microbiota to be low in both bacterial diversity and abundance [17]. Although, the ocular microbiota diversity was lower than the gut and fecal microbiotas of cattle, the bovine ocular microbiota was higher in diversity in comparison to what has been identified in the human ocular microbiota [17, 22]. However, the only other study investigating the bovine ocular microbiota reported high alpha-diversity [2] similar to diversity observed in cattle rumen and feces. This observation was inconsistent with the bovine ocular microbiota identified in this study as diversity estimates were 75–100 times lower than the estimates reported by Cullen et al. (Fig. 2) [2]. However, the study by Cullen et al. used a single time point analysis and therefore have not investigated the changes in the ocular microbiota over time [2]. As such, to our knowledge this is the first report of the changes and re-establishment of the ocular bacterial community over time.

### Core taxa present within the bovine ocular bacterial community

The core microbiota analysis identified three ASVs to be part of the CMM that accounted for a major proportion of the reads sequenced (Figure S3). The three core ASVs identified belonged to the potential opportunistic pathogen genus *Mycoplasma*, family *Weeksellacea,* and the family *Pasteurellaceae.* In a study conducted by Haung et al. [29], the conjunctiva of adult humans were sampled and genera related to *Corynebacterium* and *Pseudomonas* were identified to be the primary bacteria found among samples and concluded that bacteria found in the eye are typically deemed as opportunistic pathogens. Family *Weeksellacea*, is a polyphyletic family and as the ASV identified cannot be classified further, it is difficult to predict the role of this family in the bovine ocular microbiota [30]. *Pasteurellaceae* is a ubiquitous family found in many locations of the body including, rumen, skin, lower gut. Certain members of the *Pasteurellaceae,* such as *Pasteurella multocida* are opportunistic pathogens of many animal species and are a causative agent of bovine respiratory disease [13]. Therefore, many roles have been associated with the family *Pasteurellaceae*. The list of ASVs belonging to the CMM with average relative abundance is reported in Supplementary S11.

In addition to identifying core bacterial taxa present within all-time points to identify possible autochthonous bacterial species within the ocular microbiota, we identified core bacterial taxa present within each time period sampled. This analysis identified 54 unique ASVs across the four sampling periods. Interestingly, 51 of these ASVs belonged to Phylum *Firmicutes*, *Bacteroidetes*, or *Proteobacteria*, which accounted for 79% of the core group. Of the three major phyla making up this core group, the majority belong to opportunistic pathogen groups, suggesting that the core bacterial community within the bovine ocular microbiota may harbor opportunistic pathogens which may respond to environmental changes and physical damages that lead to IBK and other eye related diseases. This observation of low diversity and predominance of *Firmicutes* coincides with observations reported in the human ocular microbiota where commensal opportunistic pathogens, such as *Staphylococcus*, were reported to dominate [17, 22]. Additionally, we also identified ASVs belonging to genus *Staphylococcus* further supporting the observation of predominance by opportunistic pathogens.

Analysis of core ASVs shared across the different time points revealed that out of the 29 core ASVs identified in the baseline sampling, only 2, 4, and 13 of these ASVs overlapped with bacterial communities identified on days 21, 41 and 139, respectively. Similarly, 3, 5, and 40 ASVs accounting for 79, 80, 84% of the total reads for each time point were identified on days 21, 41, and 139, respectively. Hierarchical clustering of these core ASVs based on abundance displayed clear clustering based on time point where days 21 and 41 clustered closely together whereas, sampling on days 0 and 139 clustered together away from samplings on days 21 and 41 (Fig. 3). The major bacterium observed in this difference included *Pasteurellaceae, Mycoplasma,* and *Moraxella*. This shows that once the bovine ocular microbiota is disrupted the opportunistic pathogens predominate decreasing the diversity and predisposing animals to IBK and other infections.

While a CMM was identified, we sought to assess what taxa may be different among the animals at each sampling time point. As such, we used DESeq2 [31] to find the top 50 different ASVs comparatively against each sampling (i.e. 0d vs 21d, 0d vs 41d, etc). Difference were seen in taxa from phyla Firmicutes and Proteobacteria which displayed higher abundance on days 0 and 139. Interestingly, ASV_2_Mycoplasma, known as one of the CMM taxa, was also present in the differential ASV subset cohort. As such, this taxon still showed high abundance among all four samplings (Supplementary S4).

Similar to the global changes observed based on taxonomic changes, the predicted functional genes also displayed similar clustering of samples. This suggests that, in the bovine ocular microbiome, taxonomic changes are reflective of functional changes. However, further investigation using shotgun metagenome sequencing is needed to accurately demonstrate if taxonomic changes always result in functional changes.

### Shifts in the ocular bacterial community in response to IBK

The study was designed to evaluate microbiota composition changes over time in a cattle cohort. However, we identified a small subgroup of cattle, consisting of 19 individuals, that were infected with IBK and underwent treatment. There were significant differences in bacterial community composition between ulcer score classifications (Supplementary Table 1). This suggests changes in the bacterial community is associated with size of ulcer and in-turn disease severity. Additionally, the PCoA plot displayed significant clustering based on timing (pre or post) relative to IBK infection (Fig. 5) suggesting a microbial community shift during IBK infection. A PERMONAVA analysis was utilized to assess significant differences that may be observed between pre and post infection. As such, findings showed a significant difference of *p* = 0.003. Additional investigation of the abundance of *Mycoplasma* and *Moraxella* pre and post IBK infection demonstrated *Mycoplasma* abundance to significantly increase (*p* < 0.001) in samples collected after IBK infection and *Moraxella* abundance to significantly decrease (*p* < 0.001) in samples collected after IBK infection further implying that these two genera may play a role in IBK infections. Interestingly in all 19 animals, the ulcers were identified 21–41 days post perturbation of the eye. As such, it is tempting to speculate the susceptibility window for IBK infection may be within 21 days post irritation of the eye. The age of the calves that were infected with IBK ranged from 62 to 120 days at the onset of infection. However, since animals (*n* = 19) were treated for IBK once identified, the change identified could also be in response to the antibiotic treatment. Due to the small number of animals infected in this study we were unable to further pursue this question as to which features in the microbiota may be indicators of IBK infection or may predispose animals to IBK.

### *Mycoplasma* and *Moraxella* are common inhabitants of the bovine ocular microbiota

Current literature suggests members of the genus *Moraxella* and *Mycoplasma* to be associated with IBK, which then may allow *Moraxella bovis* to cause disease [16]. We identified both *Moraxella* spp*.* and *Mycoplasma* spp*.* during all time points sampled, suggesting that at least some members of these two genera are likely commensals of the bovine ocular microbiota. The reason for the overall *Moraxella* spp*.* decrease in prevalence with a relative increase in *Moraxella bovoculi* observed on day 139 is not known but may be the result of multifactorial pressure consisting of a combination of immune maturation, environmental, and/or vaccination status among other things [10, 27, 28]. Schnee et al. [16] reported herds with high abundances of *Mycoplasma bovoculi* to show a higher prevalence of acute IBK as this pathogen could be facilitating infection by *Moraxella* spp*.* This group also reported higher abundance of *Moraxella* spp*.* in IBK infected herds compared to non-infected herds. Similar to this study, Cullen et al. [2] observed *Moraxella* to be one of the most abundant genera of bacteria present in the bovine ocular microbiota.

Previous reports in the human ocular microbiota have suggested the IgA levels in the eye to affect structuring of the ocular microbiota [17]. We investigated the effect of IgA on ocular microbiota using ocular swab samples from animals with significantly different ocular bacterial communities. IgA concentrations did not differ in animals with different bacterial community composition, suggesting IgA concentration was not a driver of the ocular microbiota in this study (Figure S6). However, this does not preclude other immune parameters that were not assessed, such as cell mediated immunity from potentially affecting microbial community structures [27, 28].

The study was originally designed to test vaccine efficacy against pink eye. Although the vaccine treatments did not have a significant effect on the ocular bacterial community, it is possible that the treatment effects on the establishment of the bacterial community were not null. Cattle in this study also received an industry standard set of routine vaccinations for viral diseases, clostridial diseases, and parasiticides that may have caused perturbation. Additionally, the cattle used in this study were not the same age at sampling (Figure S12) however, age was fitted in the model and variation due to age was accounted in the model. This was also clear from the significant effect of age seen in the beta-diversity analysis. Our observation is consistent with a study performed by Wen et al. [32] who reported that the human ocular microbiota changes with age. As such, this study also lends information into the establishment of the ocular microbiota with age. Data from this study suggests the ocular microbiota establishes quickly, but recovery after perturbation is slow and could take between 21 to 98 days. As such, future investigations of the microbiota need to be longitudinal. Additionally, this study also demonstrates that given the bovine ocular microbiota is susceptible to change, microbiota manipulation could be used to introduce beneficial microbes to establish a more stable and resilient microbiota.

## Conclusion

The understanding of the establishment and composition of the ocular microbiota in cattle is limited. In this study, to better assess bacterial composition changes within the bovine eye, we performed a longitudinal study to evaluate bacterial community changes over time of the ocular microbiota using 16S rRNA sequencing. Our results demonstrate that the ocular microbiome in calves is similar between the eyes and the perturbation of one eye results in similar changes in the other eye. Additionally, we show that the bovine ocular microbiota has higher diversity than the human ocular microbiota and is predominated by opportunistic pathogens and does not appear to be influenced by overall IgA levels. We report that the bovine ocular microbiota is slow to recover after perturbation and as such, provide opportunity for opportunistic pathogens to colonize and cause disease. Additionally, many factors including age, time, and ulcer infection affect the bovine ocular bacterial microbiota composition.

## Methods

### Animals

All animal-related procedures and interventions implemented in this study were approved by the Institutional Animal Care and Use Committee (IACUC #1774) at the University of Nebraska-Lincoln.

### Study 1

The experimental population consisted of calves (*n* = 227) with three treatments of Autogenous (Phibro Animal Health) (*n* = 80 animals), Commercial (OCU-GUARD MB-1, Boheringer Ingelheim) (*n* = 77 animals), and Placebo (Emulsigen®-DL 90 adjuvant, Phibro Animal Health) (*n* = 70 animals) designed to evaluate the effectiveness of different vaccination formulations for pink eye. Treatments were randomized within cohort of both intact male and female as well as breed composition. Breed composition was defined as purebred Angus, black Simmental composites, or red Simmental composites where the composites contained varying percentages of Simmental, Angus, and Red Angus. Average age of calves on trial on day 0 was 65 days ±15.02 and was 204 ± 15.11 days at weaning at the end of the sampling period (day 139). Calves were born between mid-February and mid-April and remained nursing on dam throughout the duration of the trial. All cattle on trial remained within the same facility at the University of Nebraska-Lincoln ENREC warm season pasture without access to supplemental feed. Four sampling time points were included in the study and eye swabs were obtained on days 0, 21, 41, and 139 (Figure S1). On day 0, a sample was collected before giving the treatment. The treatments were given on day zero and a booster was given on day 21 after swabbing the eye to collect the day 21 sample (Figure S1). During the study there were 10 deaths and 2 animals were removed due to health issues, resulting in 227 animals sampled. Additionally, 19 animals were not sampled during different time periods due to heat stress. Calves were evaluated daily by trained farm staff for the presence of lesions consistent with IBK. These include excessive tearing, ulcers, or photophobia responses by calves. Farm staff were trained by the authors to recognize IBK lesions and were given scoring cards with punches ranging from 1 to 6 mm in diameter so that they could estimate lesions size and severity. Out of the 227 calves on trial, 19 became infected with IBK. Calves with IBK lesions were treated systemically with Florfenicol, florfenicol plus flunixin meglumine, oxytetracycline, or tulathromycin (Nuflor, Resflor, LA 300, or Draxxin, respectively) based on treatment guidelines provided by the attending veterinarian, using label dosages and routes. All IBK infected calves were treated once with an antibiotic intervention except for one animal that was treated twice due to the severity of the infection. The calf treated twice was removed from the analysis of pre- and post- IBK community changes, and only samples collected after 21 days of treatment were used for this analysis. After systemic antibiotic treatment, the calves were returned to the main herd on pasture.

### Study 2

An experimental population consisting of 30 heifers were used to evaluate the effect of perturbation of one eye on the bacterial community composition of the other eye. The experimental design included, swabbing of both the left and right eyes in 10 animals on day 0, swabbing of the left eye in 10 animals on day 0, and swabbing of the right eye in 10 animals on day 0, followed by swabbing of left and right eye all animals for a second time 17 days later (*n* = 30). All swabs collected were processed as described above and 16S rRNA sequencing and analysis was performed as described below.

### Ocular regional samplings

Calves were placed in a hydraulic squeeze chute and the head was manually restrained while the eyelids were opened by trained animal care personnel and the cornea and conjunctival tissues were vigorously swabbed using a single eSwab® collection swab (Copan Diagnostics, Murrieta, CA). Care was taken to ensure the swab only touched the ocular and conjunctival surfaces by trained staff wearing gloves. Sample collection personnel were the same throughout the study to avoid any sampling bias. Additionally, all calves were swabbed on the same side (right or left) at each given time sampling. At each sampling, the eye sampled was alternated. The swabbing can be considered as a perturbation of the ocular microbiota. All swabs were placed in Amies transport media and immediately placed in a cooler for transport to the laboratory where an aliquot was frozen and stored at − 80 °C awaiting molecular analysis of the microbiota. Additionally, for identifying *Moraxella* spp*.* in the samples, the tubes of media were vortexed for 5–10 s prior to using the original swab to streak the media on tryptic soy agar with 5% sheep blood (Remel, Lenexa, KS). Plates were incubated at 37 °C in 5% CO_2_ for 24 h. After 24 h, suspected *Moraxella* spp*.* were chosen based on colony morphology and oxidase positive status that were re-streaked on blood agar and incubated for 24 h prior to MALDI-TOF MS analysis.

### MALDI-TOF mass spectroscopy identification

MALDI-TOF MS identification was performed using the smear method recommended by the manufacturer and that has been previously described [33]. Briefly, several colonies were dissolved in 300 μl of HPLC water and 900 μl of absolute ethanol was added to the mixture. The solution was centrifuged (2 min at 16,000 x g) and the supernatant decanted before allowing the sample tubes to air dry. The pellet was then dissolved in 25 μl 70% formic acid and 25 μl of acetonitrile and centrifuged again (5 min at 16,000 x g). Lastly, 1 μl of the sample was placed onto the target plate and allowed to air dry after which 1 μl of α-cyano-4-hydroxycinnamic acid matrix solution (Bruker Daltonik, Billerica, MA) was placed onto the well. When the wells were completely dry the plates were analyzed by MALDI-TOF MS using a MALDI Biotyper (Bruker Daltonik) in positive linear mode with a mass range of 2–20 kDa m/z and a laser frequency of 60 Hz. Machine calibration was performed using Bacterial Test Standard (Bruker Daltonik). Biotyper software (Bruker Daltonik) was used to compare the obtained spectra against a large database to identify the isolates to the species level. According to the manufacturer’s recommendations, scores of > 2.00 were considered as positive bacterial identification.

### DNA extraction and sequencing

DNA was extracted using Lucigen Quick Extract kit (Lucigen Corporation Middleton, WI) following the manufacturer’s instructions with the following modification. Briefly, a bead beating step using a TissueLyser (Qiagen Inc., Valencia, CA, USA) at a frequency of 20 Hz for 15 min prior to water bath incubations at 65 °C or 6 min and 98 °C for 5 min was implemented. The quality of the DNA extracted was assessed using gel electrophoresis and the resulting good quality DNA was stored at − 20 °C until used for bacterial community analysis. Amplification of the V4 region of the 16S rRNA gene and sequencing using multiplexed primers was performed using the MiSeq- 250 bp paired end sequencing strategy as described previously [34]. Briefly, barcoded universal primers specific to the V4 region were used to amplify the 16S rRNA gene in 25 μ l PCR reactions. Library preparation PCR reaction contained 0.75 Units of Terra PCR Direct Polymerase Mix (Clontech Laboratories Inc., Mountain View, CA, USA), 1X Terra PCR Direct Buffer (Clontech Laboratories Inc., Mountain View, CA, USA), 0.4 uM indexed primers, and 20-50 ng of DNA. Amplifications were performed on a Veriti 96-Well Thermocycler (Life Technologies, Carlsbad, CA, USA) with conditions of 98 °C for 2 min followed by 25 cycles of 98 °C for 30s, 58 °C for 30 s, 68 °C for 45 s, with a final extension of 68 °C for 4 min. The resulting amplicons were normalized using the NGS Normalization 96-Well Kit (Norgen BioTek Corp., Thorold, ON, Canada) according to manufactures instructions. In short, half of the samples were switched from using the Charm Biotech normalization kit in order to retain a higher amplicon concentration. Samples normalized using the Charm Biotech kit were pooled together and purified using NucleoSpin® Gel and PCR Clean-up (Macherey-Nagal Inc., Bethlehem, PA, USA) according to manufactures protocol. Concentrations of pooled samples were confirmed using Denovix Fluorescence High Sensitivity Assay (Denovix Inc., Wilmington, DE, USA). Ready to sequence libraries were further analyzed using the Agilent BioAnalyzer 2000 (Agilent Technologies, Santa Clara, CA, USA) and sequenced using the 250 bp paired end sequencing strategy using the Illumina MiSeq platform with V2 500 cycle sequencing kit as described by the manufacturer (Illumina, San Diego, CA, USA). Each sequencing run included at least 4 negative controls and were sequenced and were used to remove any reagent associated contaminants as described below. Twenty-seven samples were removed from the study post DNA extraction due to poor DNA quality and sequence read depth.

### Protein quantification and ELISA preparation

A subset of animals (*n* = 21) were identified based on microbial community differences that reflected the largest change in community structure across the days sampled with swabs representing all sampling time points. The swabs from this subset of animals were used to evaluate bovine immunoglobulin A levels (IgA). Briefly, protein levels in the eye swabs were quantified using Qubit™ Protein Assay Kit (Invitrogen Carlsbad, CA, USA) according to the manufactures protocol and normalized protein amounts (19–24 mg) were assayed using the Bovine IgA ELISA kit (BioMatik Corporation Cambridge ON, Canada) according to the manufactures protocol to evaluate bovine IgA amounts.

### Data processing

Detailed bioinformatic analysis information including step by step analysis instructions has been provided as a R-markdown file in the Fernando Lab Github page below to reproduce all data and figures in this study. The bioinformatic analysis used including the mapping file, scripts, and commands can be found in the GitHub page of the Fernando Lab (https://github.com/FernandoLab/IBK-Year1-). In brief, the DADA2 pipeline [35] was used for subsequent analysis. Analysis was performed using the DADA2 pipeline in order to limit fewer false positive sequences. As such, amplicon sequence variants (ASV) were used rather than operational taxonomic units (OTU). Analysis steps were performed using R v1.1.463 (R Core Team, 2019) using the phyloseq package v1.26.1 [36]. Briefly, low quality reads were filtered (Q score of ≥30) and reads were trimmed to 253 bp length. “Unique sequences” were identified by combining identical sequences and error rates were estimated to evaluate read quality. Forward and reverse reads were assembled to generate contigs for the V4 region. Furthermore, quality filtering was performed to remove sequences with ambiguous bases, incorrect contig length, assembly, or chimeras. SILVA reference alignment database v138 was used to assign taxonomy and a phylogenetic tree was generated using MOTHUR (v.1.42.1) [37]. The resulting phylip.tree, sequence table, mapping file and taxonomy table were used to generate a “phyloseq object” which was used for further analysis.

Negative controls sequenced were used to remove any potential contaminant ASVs arising from reagent contamination and were removed using the decontam package as described previously [38]. Additionally, ASVs identified only in one sample were removed.

All sequencing data generated in this project have been deposited in the NCBI sort read archive under accession number PRJNA600014 & PRJNA690108. Detailed information regarding the bioinformatic analysis used including the mapping file, scripts, and commands can be found in the GitHub page of the Fernando Lab (https://github.com/FernandoLab/IBK-Year1-).

### Predicted functional gene analysis

Representative sequences for each ASV were converted into a “biom” file for subsequent analysis using the program Phylogenetic Investigation of Communities by Reconstruction of Unobserved States (PICRUSt2) [23]. Briefly, fasta files were aligned to reference tree HMMR [39–41] and genes were predicted that contained the nearest-sequenced taxon index. Using the metagenome_pipeline.py command, read depth per ASV were divided by the predicted 16S copy numbers and the ASV read depths were multiplied by the predicted gene family copy number per ASV [42] to obtain abundance of each predicted gene. This information was utilized to assess the metagenome of the microbial communities. MinPath [43] was used to identify and infer pathway level abundances. Resulting KO outputs were read back into ‘R’ and a phyloseq object was created for subsequent analysis.

### Statistics

Statistical analyses were performed using the R package ‘vegan’ [44] using the adonis function. The PERMANOVA analysis was performed using the weighted UniFrac distance matrix to identify factors affecting microbial community structure. The model used for PERMANOVA analysis included the main effects of sampling time point, age, treatment, hide color, breed, ulcer presence, and sex. Alpha diversity was evaluated using observed ASVs and Chao1 values and statistical significance was evaluated using a pairwise Wilcoxon rank sum test within ‘R’ [45]. PERMANOVA analysis for bacterial community changes in pre- and post IBK infection was evaluated using a weighted UniFrac distance matrix. Additionally, a t-test was performed to evaluate significant changes in *Mycoplasma* and *Moraxella* spp. abundance before and after IBK infection [46]. No treatment effects were detected on IBK occurrence. DESeq2 was utilized to identify differential ASVs and predictive genes (KOs) present between timepoints using the Benjamini error correction method [31]. PERMANOVA analysis for predicted functional genes were used based off a Bray-Curtis distance matrix. All significance was determined at *p* < 0.05 except for differential KOs where a threshold of *p* < 0.01 was used.

## Supplementary Information

**Additional file 1: Figure S1.** Experimental design consisting of time of sampling, vaccination given, and average age of calves during each time period. A perturbation, or disruption, to the ocular microbiota occurred at each time sampling.

**Additional file 2: Figure S2.** Principal Coordinate analysis (PCoA) demonstrating between individual cohort sample variations in beta-diversity. PCoA plot was generated using a weighted UniFrac distance matrix. A) Cohort A B) Cohort B and C) Cohort C. PERMANOVA revealed there was no significant difference between the eyes sampled at day 17 (*p* = 0.37, *p* = 0.245, and *p* = 0.659; respectively).

**Additional file 3: Figure S3.** Distribution of “Core” ASVs across sampling time-points demonstrating predominance of opportunistic pathogens in the bovine ocular microbiota. Perturbations, or disruptions, to the ocular microbiota occurred by taking ocular swabs. Core bacterial ASVs were identified based on the presence of an ASV in at least 80% of all samples. Only 3 core ASVs were identified that fell into this criteria. The core ASVs include; *Pasteurellaceae* spp. (Blue), *Mycoplasma* spp*.* (Orange) and *Weeksellacea* spp. (Grey).

**Additional file 4: Figure S4.** Differential ASVs found among time sampling comparison. (Key- * denotes taxa at family level, ** denotes taxa at order level, and *** denotes taxa at class level.).

**Additional file 5: Figure S5.** Distribution of opportunistic pathogens *Moraxella* spp*.* and *Mycoplasma* spp*.* across the 4 sampling time points. *Moraxella* spp*.* and *Mycoplasma* spp*.* are considered as opportunistic pathogens that predispose animals to IBK infection.

**Additional file 6: Figure S6.** Association between IgA concentration and time the ocular swab was taken from cattle infected with IBK.

**Additional file 7: Figure S7.** Prevalence of *Moraxella* spp*.* in total (7a), as well as individually for *Moraxella bovis* (7b) and *Moraxella bovoculi* (7c) obtained from the eyes of calves at different time points. Letters denote statistically different groups (*p* < 0.05). Bars represent 95% confidence interval.

**Additional file 8: Figure S8.** Observed ASVs (A) and Chao1 estimates (B) indicating alpha-diversity differences between sampling time points. Significant differences in predicted functional gene alpha-diversity was identified between all sampling periods. After initial swabbing for baseline community, the diversity significantly decreased and recovered over time in subsequent samplings. The initial swabbing, would be deemed as the first perturbation or disruption to the ocular microbiota.

**Additional file 9: Figure S9.** Principal Coordinate analysis (PCoA) demonstrating between sample variations in predicted functional genes. PCoA plot was generated using a Bray-Curtis distance matrix. Day 0 and day 139 demonstrated similar gene structure that was different from 21 days and 41 days. Color scheme; Red – time point 1, Green - time point 2, Teal – time point 3, and Purple – time point 4.

**Additional file 10: Figure S10.** Heatmap of differential KOs associated with each sampling timepoint of collection. Only KOs that are significantly different at *p* < 0.01 is shown.

**Additional file 11: Figure S11.** List of CMM ASV with relative abundance at each time sampling. (Key- * denotes taxa at family level, ** denotes taxa at order level, and *** denotes taxa at class level.).

**Additional file 12: Figure S12.** Distribution of the calves age throughout the duration of the trial.

**Additional file 13: Table S1.** Scores of ulcers from cattle infected with IBK during the study.

## Data Availability

The dataset generated and analyzed during the study are available under BioProject accession number PRJNA600014 from the National Center for Biotechnology Information (NCBI) sequence read archive (SRA). Detailed information regarding the bioinformatic analysis used including the mapping file, scripts, and commands can be found in the GitHub page of the Fernando Lab (https://github.com/FernandoLab/IBK-Year1-).

## Abbreviations

IBK: Infectious Bovine Keratoconjunctivitis
rRNA: Ribosomal RNA
PCoA: Principal Coordinate Analysis
IgA: Immunoglobulin A
ASV: Amplicon sequence variants
CMM: Core measurable microbiota
PCR: Polymerase Chain Reaction
ELISA: Enzyme-linked immunosorbent assay
PICRUSt: Phylogenetic Investigation of Communities by Reconstruction of Unobserved States
