# Supplementary figures and images for: Longitudinal assessment of the bovine ocular bacterial community dynamics in calves
Source: Anim Microbiome. 2021 Jan 30;3:16. doi: 10.1186/s42523-021-00079-3 (PMC7847012; doi:10.1186/s42523-021-00079-3)

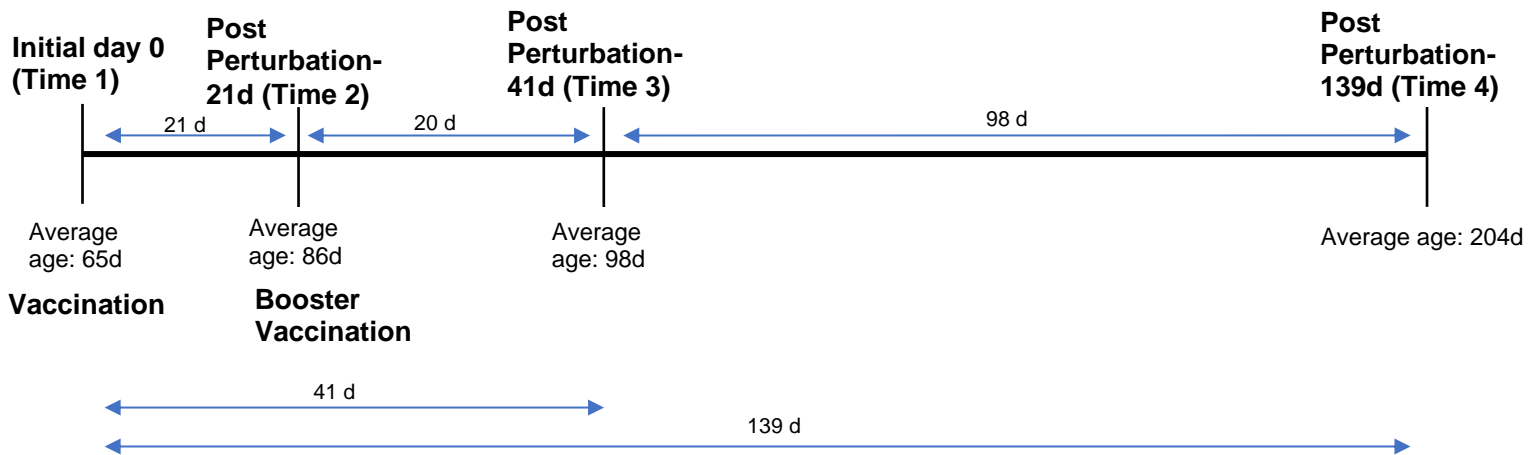

Supplement: Supplementary file 1 — Additional file 1: Figure S1. Experimental design consisting of time of sampling, vaccination given, and average age of calves during each time period. A perturbation, or disruption, to the ocular microbiota occurred at each time sampling. [file 42523_2021_79_MOESM1_ESM.pdf]

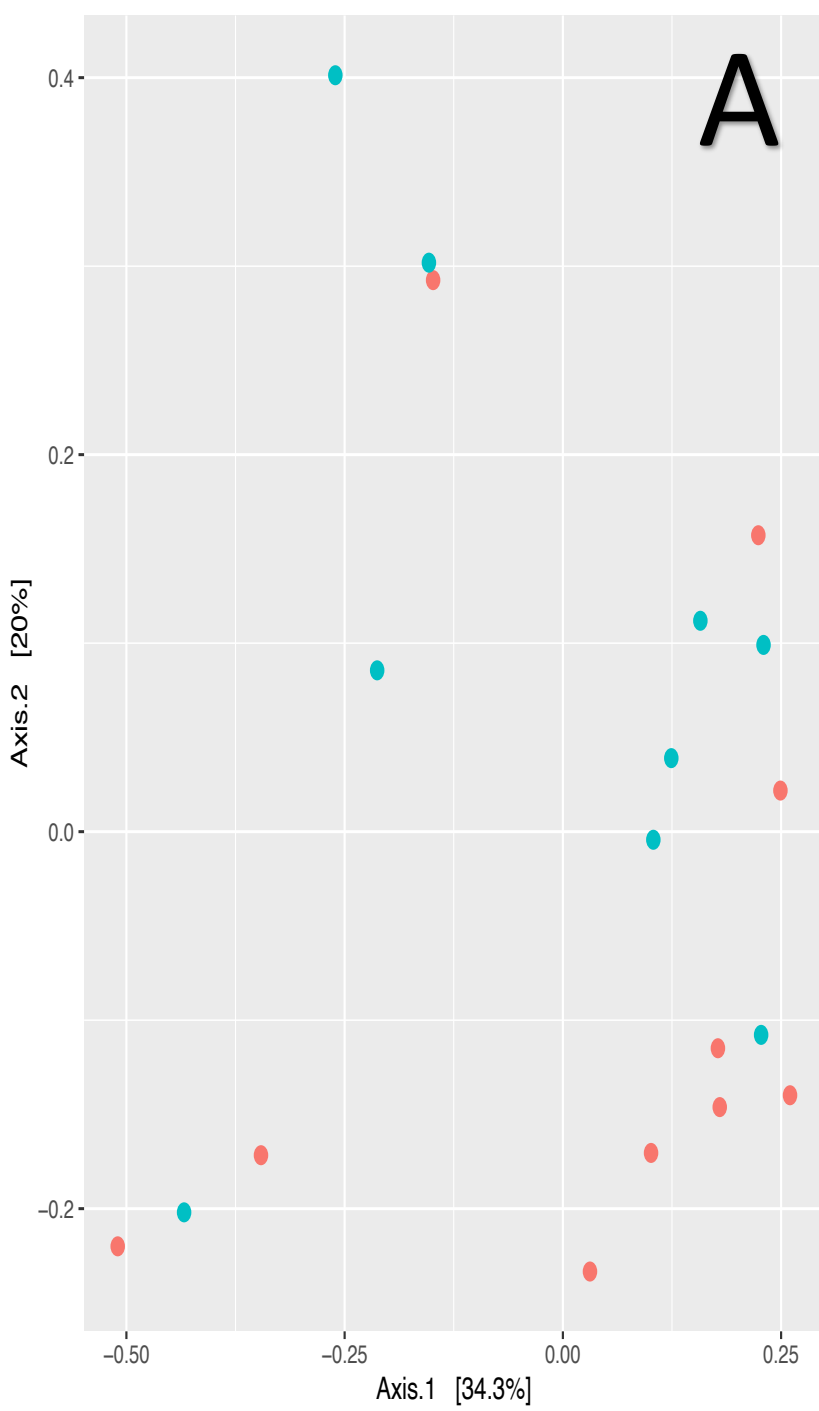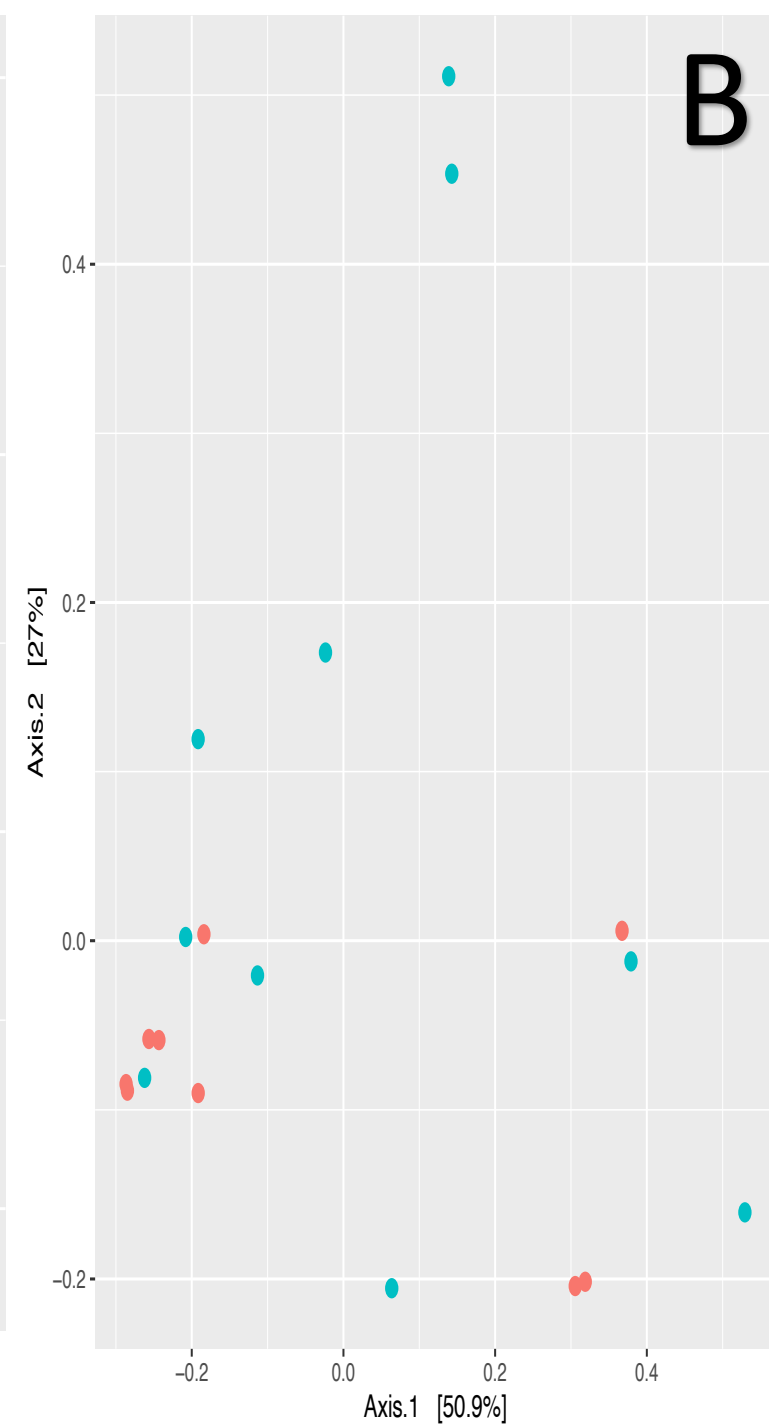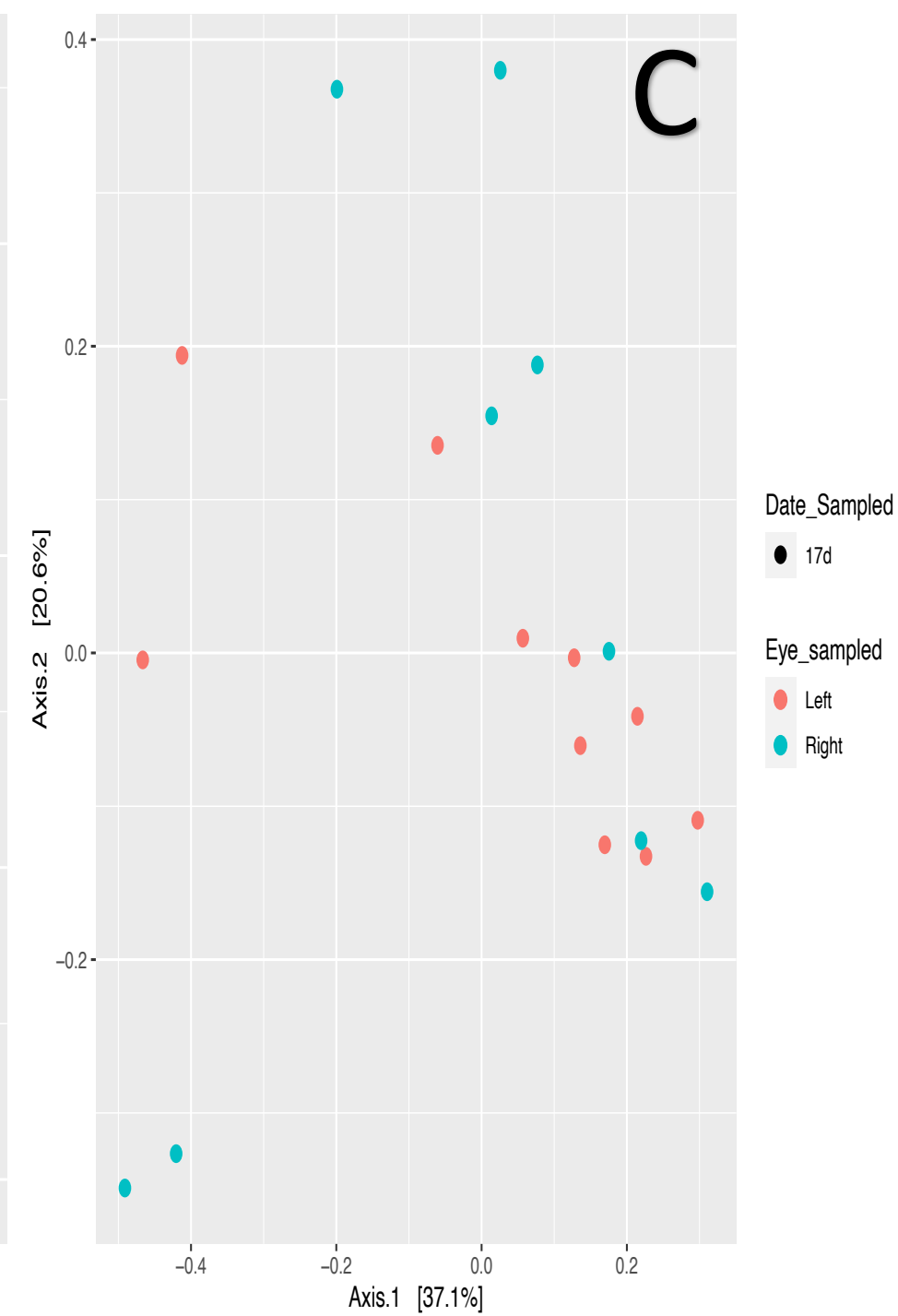

Supplement: Supplementary file 2 — Additional file 2: Figure S2. Principal Coordinate analysis (PCoA) demonstrating between individual cohort sample variations in beta-diversity. PCoA plot was generated using a weighted UniFrac distance matrix. A) Cohort A B) Cohort B and C) Cohort C. PERMANOVA revealed there was no significant difference between the eyes sampled at day 17 (p = 0.37, p = 0.245, and p = 0.659; respectively). [file 42523_2021_79_MOESM2_ESM.pdf]

## Core ASVs

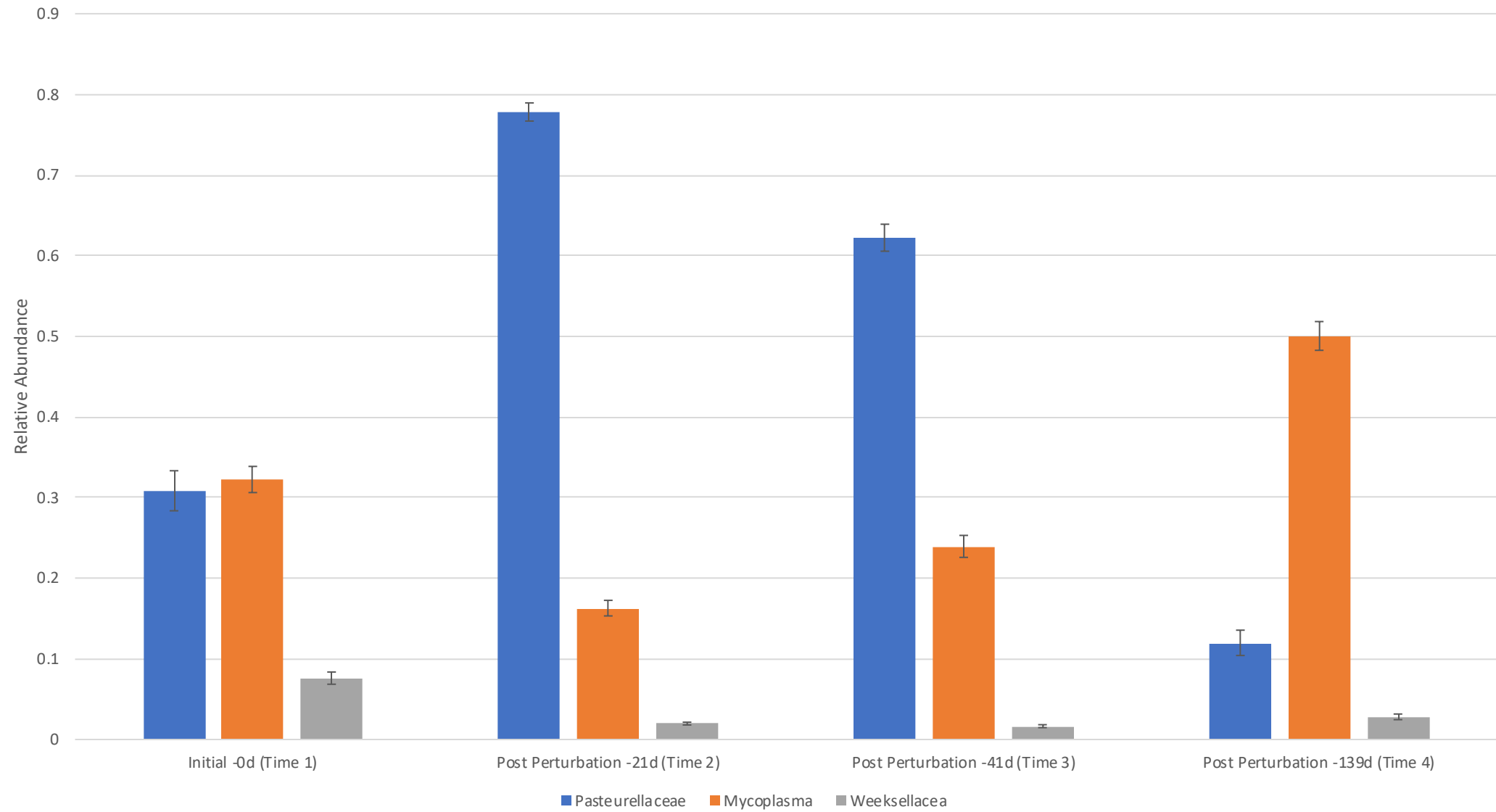

Supplement: Supplementary file 3 — Additional file 3: Figure S3. Distribution of “Core” ASVs across sampling time-points demonstrating predominance of opportunistic pathogens in the bovine ocular microbiota. Perturbations, or disruptions, to the ocular microbiota occurred by taking ocular swabs. Core bacterial ASVs were identified based on the presence of an ASV in at least 80% of all samples. Only 3 core ASVs were identified that fell into this criteria. The core ASVs include; Pasteurellaceae spp. (Blue), Mycoplasma spp. (Orange) and Weeksellacea spp. (Grey). [file 42523_2021_79_MOESM3_ESM.pdf]

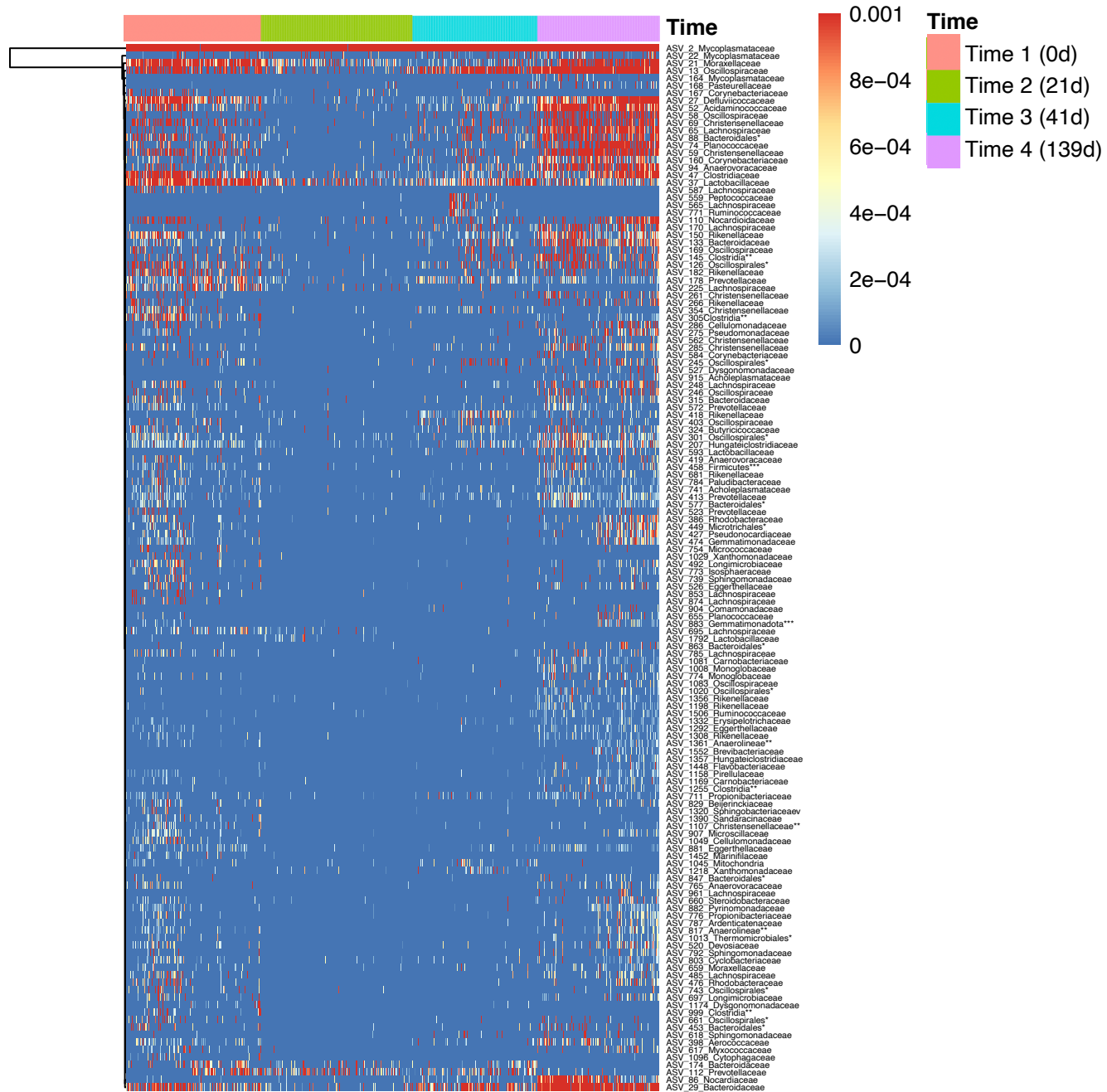

Supplement: Supplementary file 4 — Additional file 4: Figure S4. Differential ASVs found among time sampling comparison. (Key- * denotes taxa at family level, ** denotes taxa at order level, and *** denotes taxa at class level.). [file 42523_2021_79_MOESM4_ESM.pdf]

## Mycoplasma

**A**

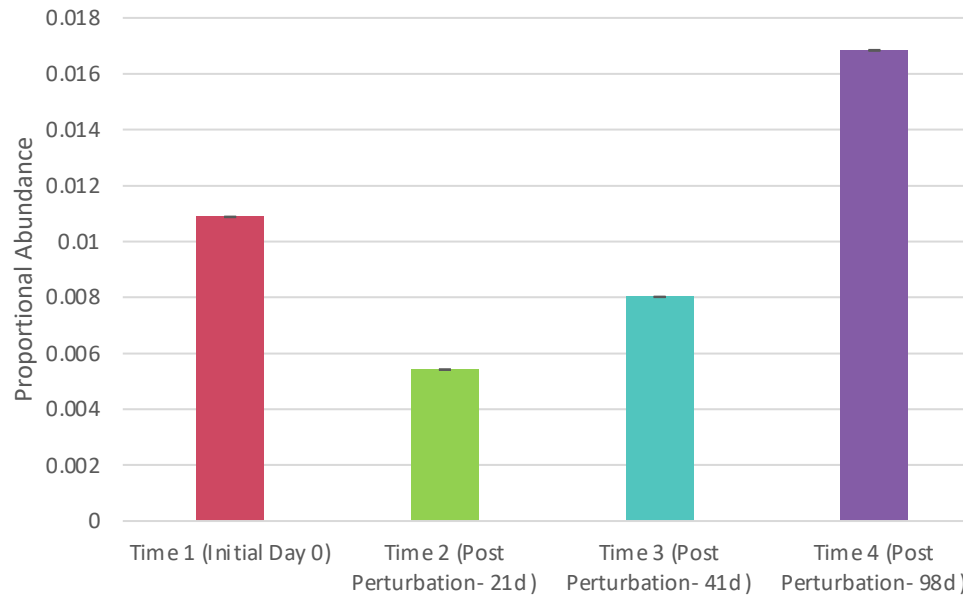

## Moraxella

**B**

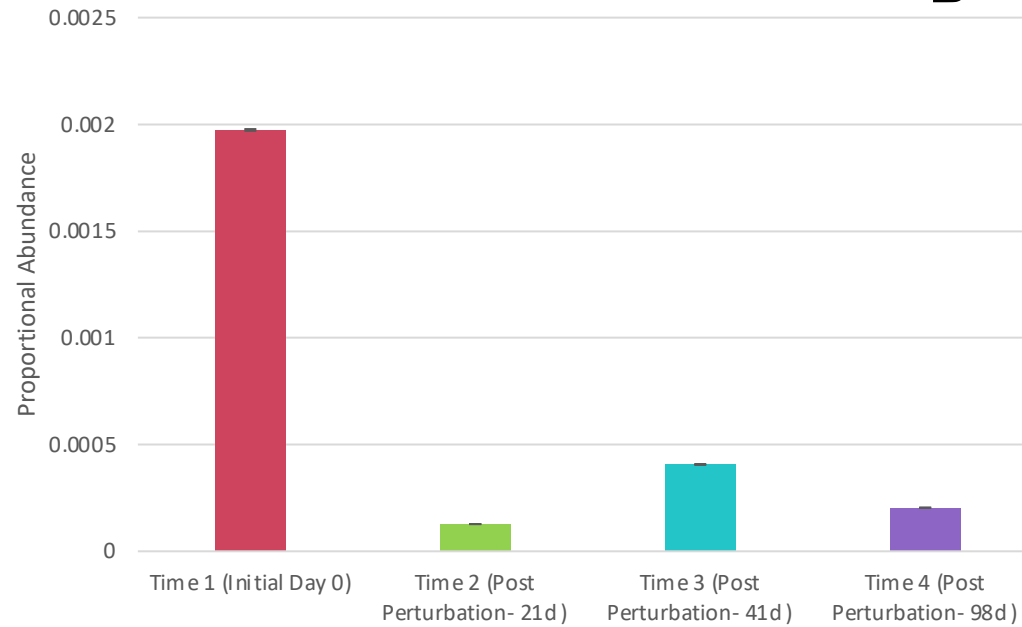

Supplement: Supplementary file 5 — Additional file 5: Figure S5. Distribution of opportunistic pathogens Moraxella spp. and Mycoplasma spp. across the 4 sampling time points. Moraxella spp. and Mycoplasma spp. are considered as opportunistic pathogens that predispose animals to IBK infection. [file 42523_2021_79_MOESM5_ESM.pdf]

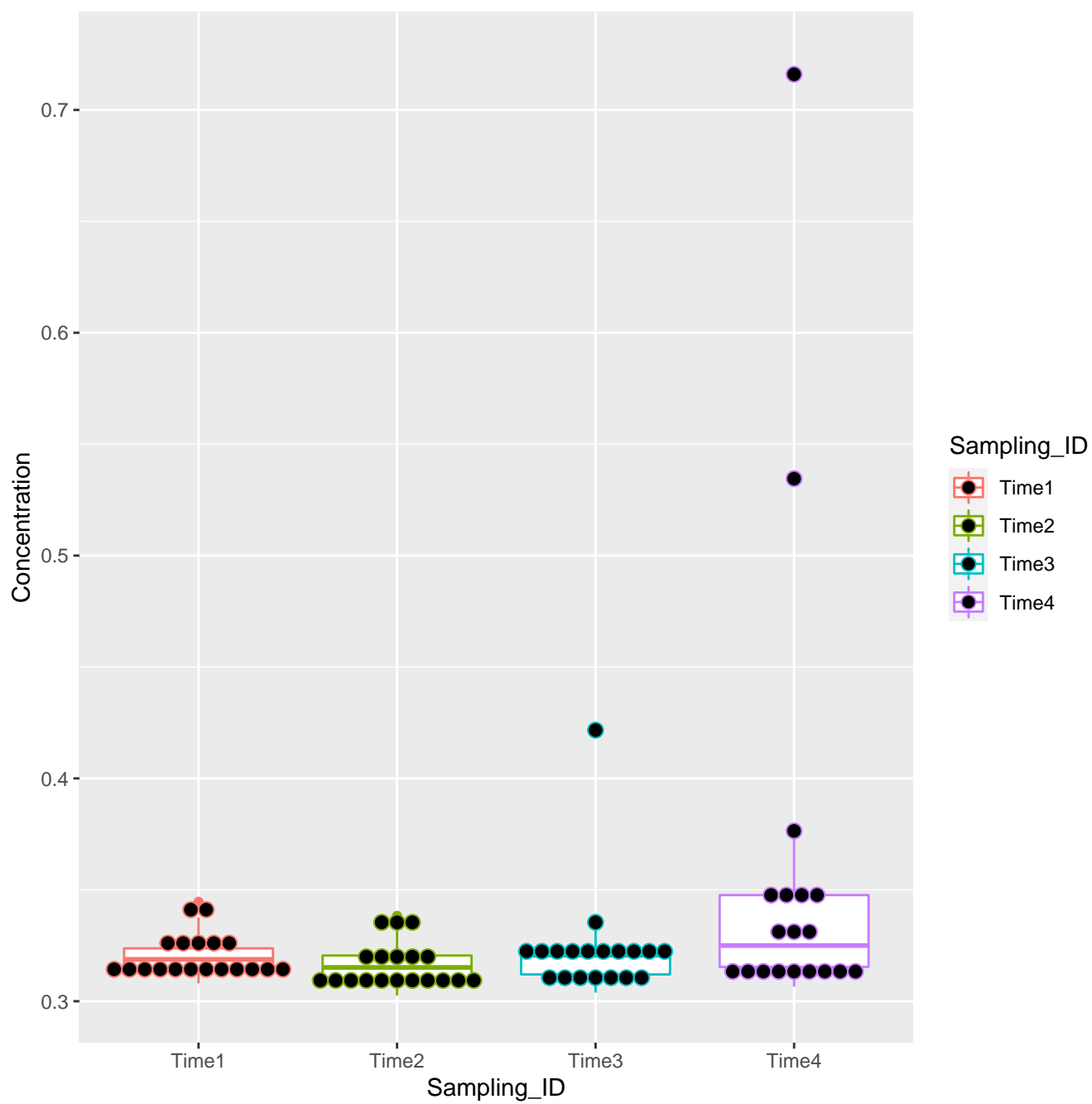

Supplement: Supplementary file 6 — Additional file 6: Figure S6. Association between IgA concentration and time the ocular swab was taken from cattle infected with IBK. [file 42523_2021_79_MOESM6_ESM.pdf]

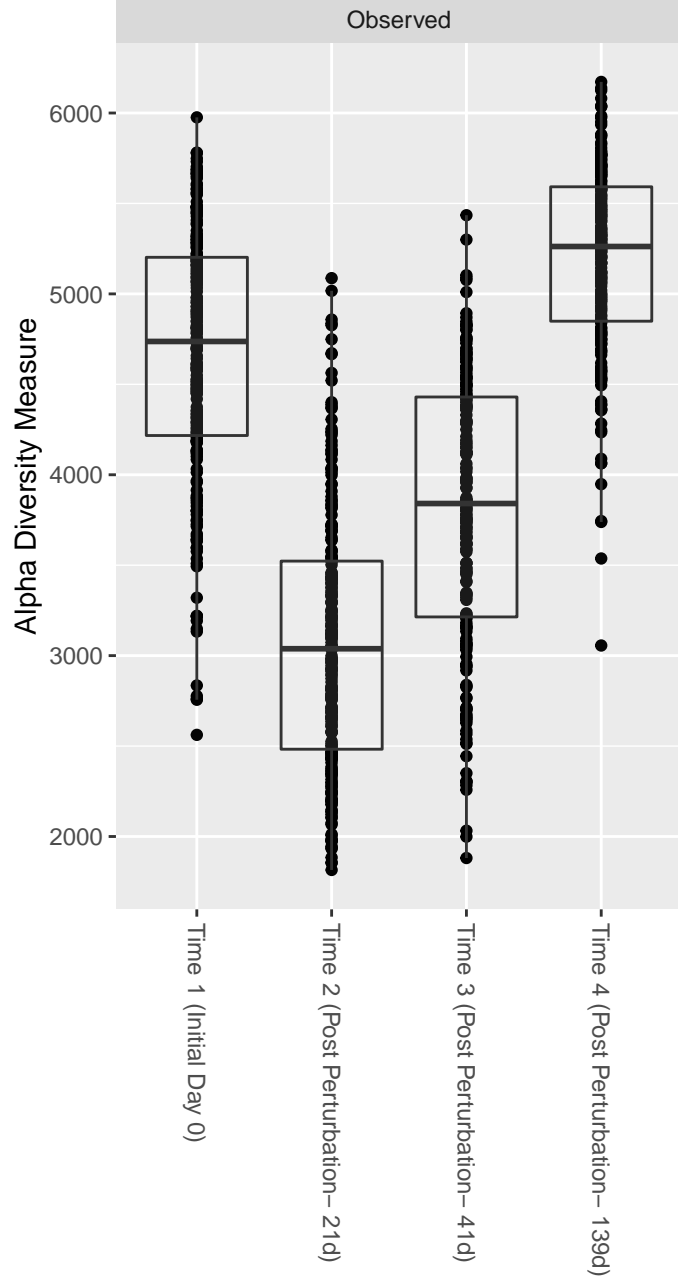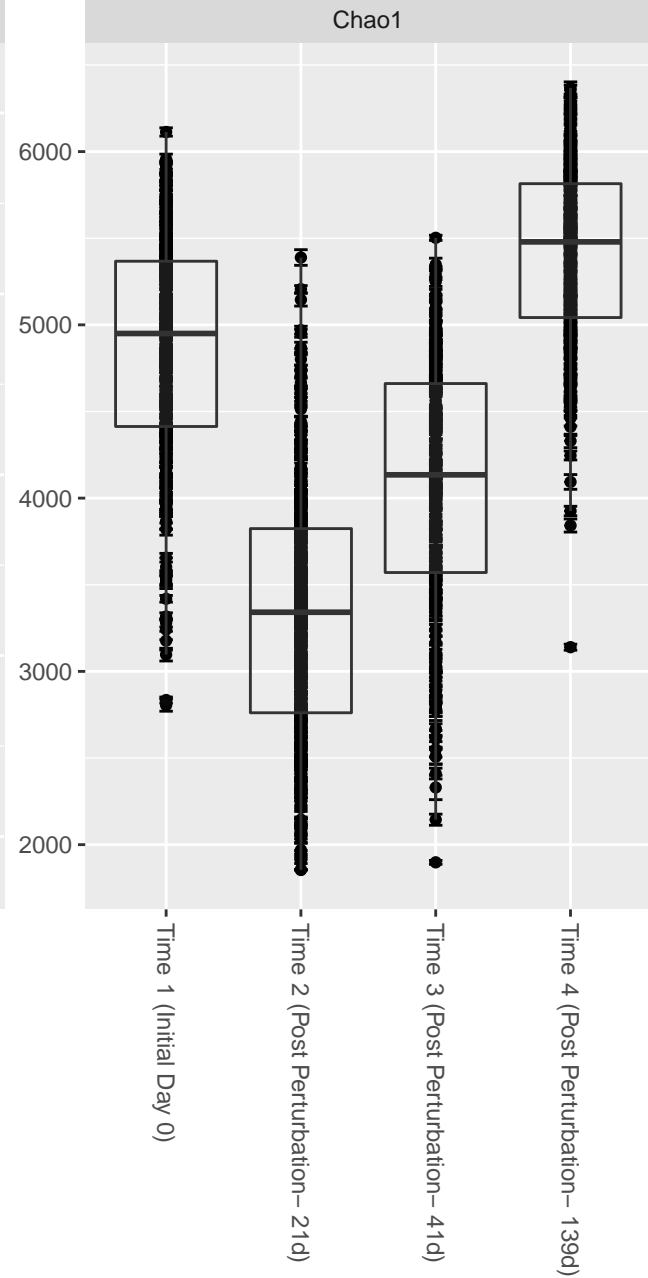

Supplement: Supplementary file 8 — Additional file 8: Figure S8. Observed ASVs (A) and Chao1 estimates (B) indicating alpha-diversity differences between sampling time points. Significant differences in predicted functional gene alpha-diversity was identified between all sampling periods. After initial swabbing for baseline community, the diversity significantly decreased and recovered over time in subsequent samplings. The initial swabbing, would be deemed as the first perturbation or disruption to the ocular microbiota. [file 42523_2021_79_MOESM8_ESM.pdf]

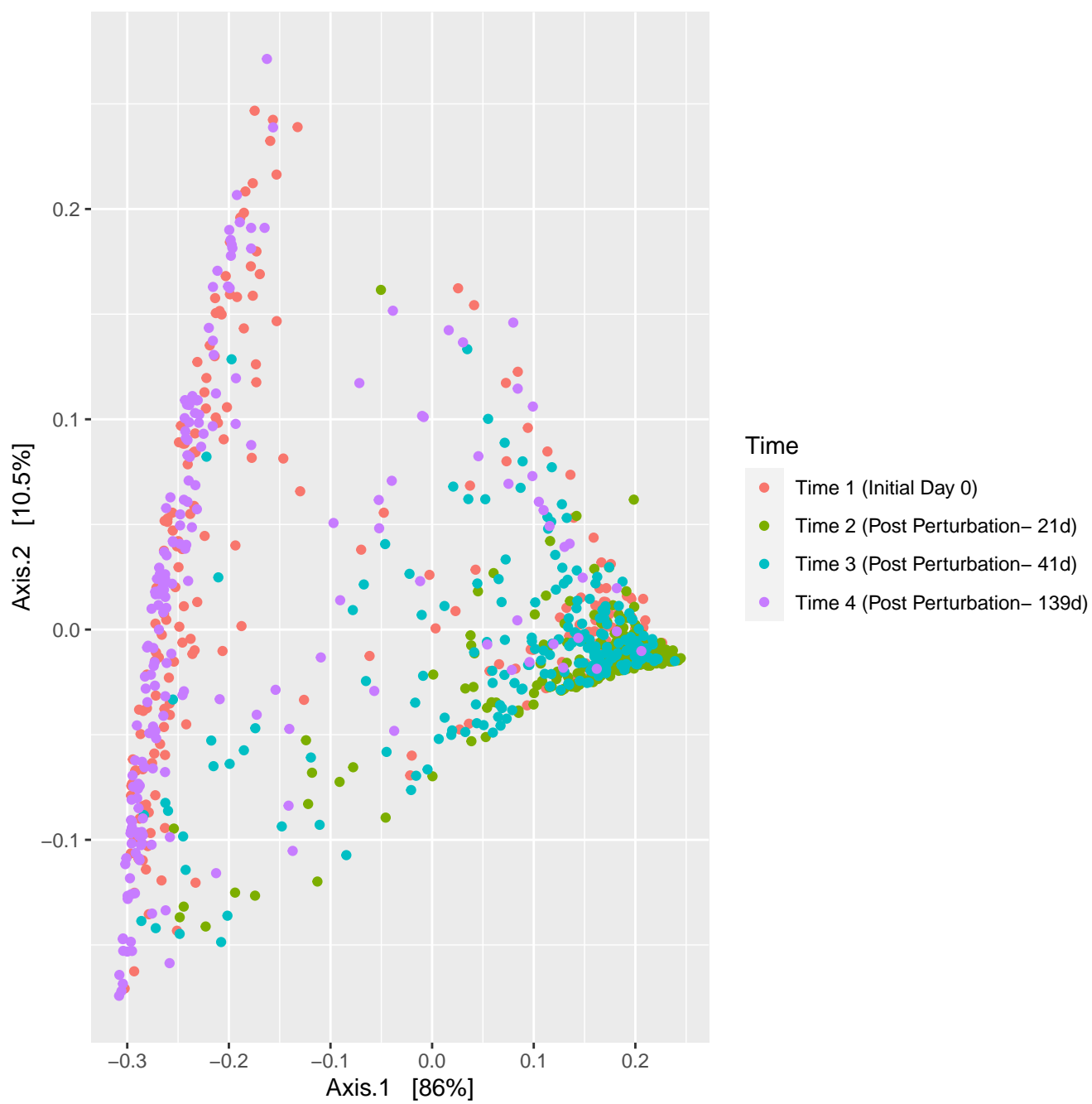

Supplement: Supplementary file 9 — Additional file 9: Figure S9. Principal Coordinate analysis (PCoA) demonstrating between sample variations in predicted functional genes. PCoA plot was generated using a Bray-Curtis distance matrix. Day 0 and day 139 demonstrated similar gene structure that was different from 21 days and 41 days. Color scheme; Red – time point 1, Green - time point 2, Teal – time point 3, and Purple – time point 4. [file 42523_2021_79_MOESM9_ESM.pdf]

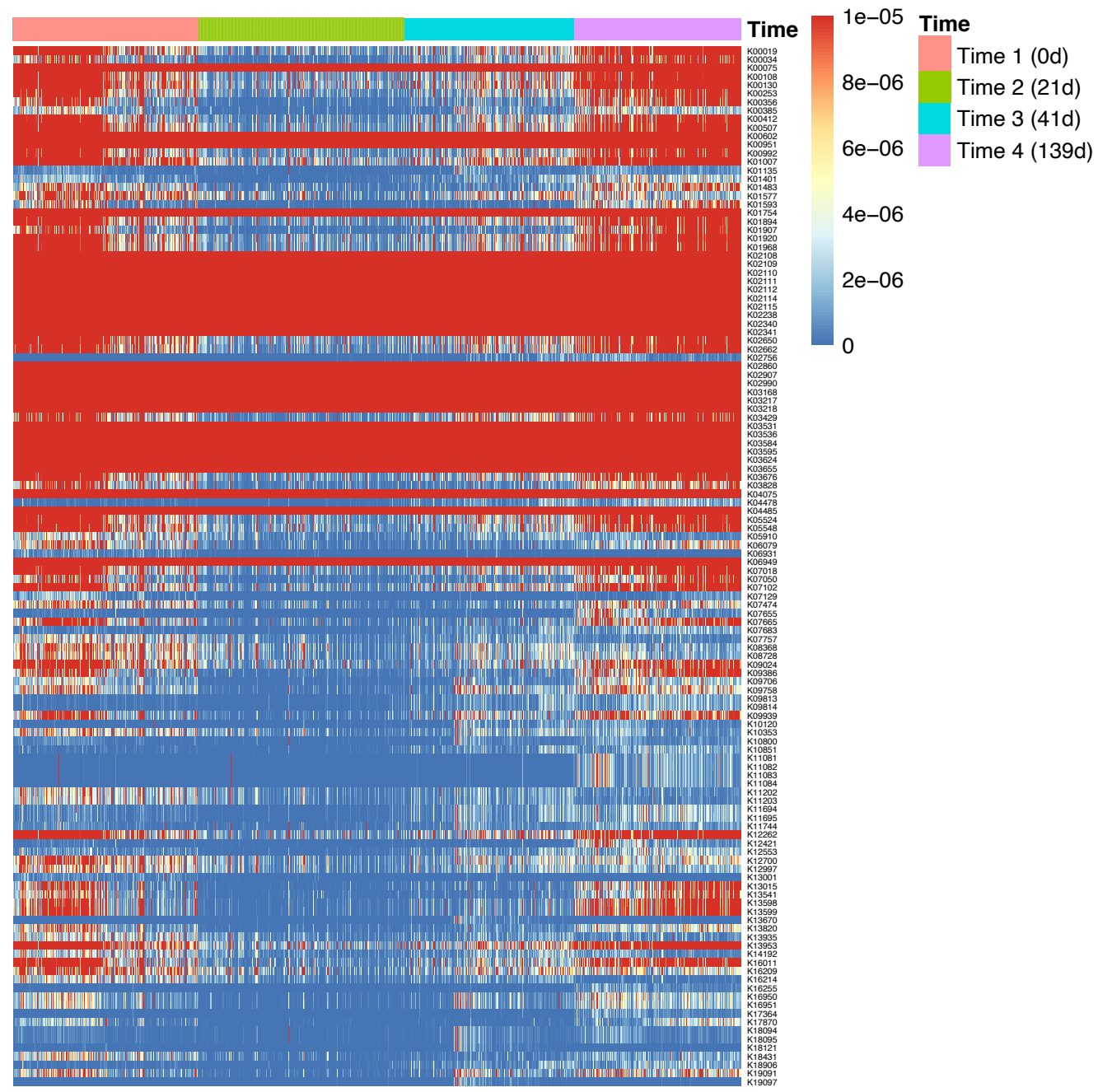

Supplement: Supplementary file 10 — Additional file 10: Figure S10. Heatmap of differential KOs associated with each sampling timepoint of collection. Only KOs that are significantly different at p < 0.01 is shown. [file 42523_2021_79_MOESM10_ESM.pdf]
