# Supplementary material for: Longitudinal assessment of the bovine ocular bacterial community dynamics in calves
Source: Anim Microbiome. 2021 Jan 30;3:16. doi: 10.1186/s42523-021-00079-3 (PMC7847012; doi:10.1186/s42523-021-00079-3)
Supplement: Supplementary file 7 — Additional file 7: Figure S7. Prevalence of Moraxella spp. in total (7a), as well as individually for Moraxella bovis (7b) and Moraxella bovoculi (7c) obtained from the eyes of calves at different time points. Letters denote statistically different groups (p < 0.05). Bars represent 95% confidence interval. [file 42523_2021_79_MOESM7_ESM.pdf]

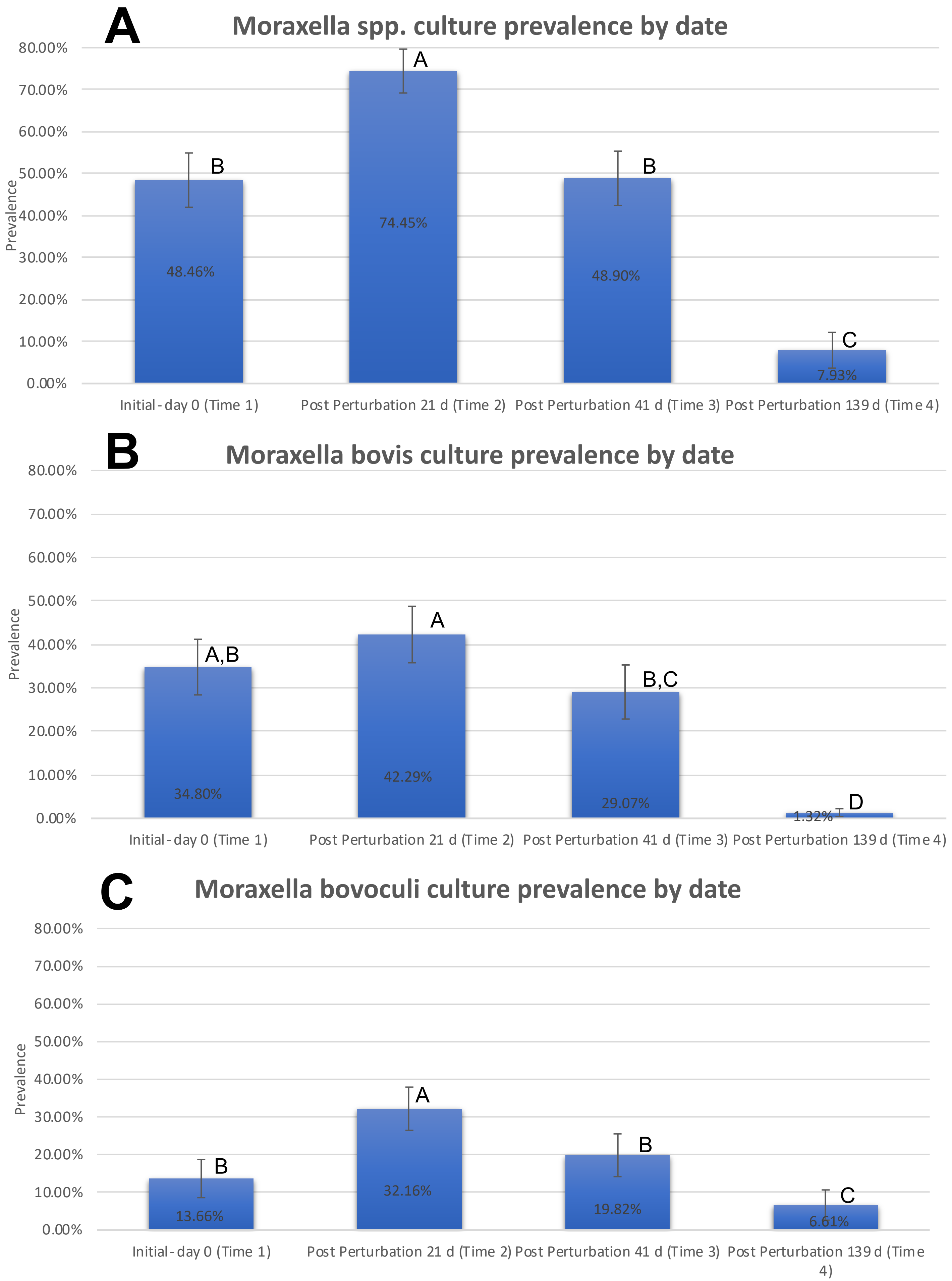
A
Moraxella spp. culture prevalence by date
Prevalence
80.00%
70.00%
60.00%
50.00%
40.00%
30.00%
20.00%
10.00%
0.00%
48.46%
B
74.45%
A
48.90%
B
7.93%
C
Initial - day 0 (Time 1)
Post Perturbation 21 d (Time 2)
Post Perturbation 41 d (Time 3)
Post Perturbation 139 d (Time 4)
B
Moraxella bovis culture prevalence by date
Prevalence
80.00%
70.00%
60.00%
50.00%
40.00%
30.00%
20.00%
10.00%
0.00%
34.80%
A,B
42.29%
A
29.07%
B,C
1.32%
D
Initial - day 0 (Time 1)
Post Perturbation 21 d (Time 2)
Post Perturbation 41 d (Time 3)
Post Perturbation 139 d (Time 4)
C
Moraxella bovoculi culture prevalence by date
Prevalence
80.00%
70.00%
60.00%
50.00%
40.00%
30.00%
20.00%
10.00%
0.00%
13.66%
B
32.16%
A
19.82%
B
6.61%
C
Initial - day 0 (Time 1)
Post Perturbation 21 d (Time 2)
Post Perturbation 41 d (Time 3)
Post Perturbation 139 d (Time 4)
