# Supplementary material for: Longitudinal assessment of the bovine ocular bacterial community dynamics in calves
Source: Anim Microbiome. 2021 Jan 30;3:16. doi: 10.1186/s42523-021-00079-3 (PMC7847012; doi:10.1186/s42523-021-00079-3)
Supplement: Supplementary file 11 — Additional file 11: Figure S11. List of CMM ASV with relative abundance at each time sampling. (Key- * denotes taxa at family level, ** denotes taxa at order level, and *** denotes taxa at class level.). [file 42523_2021_79_MOESM11_ESM.pdf]

	Initial Day 0 (Time 1)	Perturbation - 21d (Time 2)	Perturbation - 41d (Time 3)	Perturbation - 139d (Time 4)
**ASV_38_Ruminococcaceae**	0.00164253	0.00016817	0.00049397	0.00151348
**ASV_21_Ruminococcaceae**	0.00173789	0.00032892	0.00119848	0.00406054
**ASV_12_Ruminococcaceae**	0.00169576	0.00042029	0.00238153	0.00587123
**ASV_8_Ruminococcaceae**	0.00414478	0.00078978	0.00300786	0.00909132
**ASV_7_Ruminococcaceae**	0.00535765	0.00110805	0.0040702	0.00997484
**ASV_68_Ruminococcaceae**	0.00047173	5.56E-05	0.0003197	0.00172493
**ASV_74_Ruminococcaceae**	0.00188039	0.00038728	0.00017347	1.06E-06
**ASV_1_Pasteurellaceae**	0.30842976	0.77449532	0.62432583	0.113748
**ASV_3_Weeksellaceae**	0.07610735	0.0196708	0.01654275	0.0278927
**ASV_28_Rikenellaceae**	0.00106916	0.00018783	0.00091303	0.00363287
**ASV_86_Bacteroidaceae**	0.00033894	4.43E-05	0.00028393	0.00132016
**ASV_36_Bacteroidaceae**	0.00085124	0.00019146	0.0008033	0.00222232
**ASV_27_Bacteroidaceae**	0.00084179	0.00025546	0.00106123	0.00365654
**ASV_108_Prevotellaceae**	0.00115277	0.00043808	9.10E-05	4.92E-07
**ASV_57_Prevotellaceae**	0.00206076	0.00076537	5.25E-05	9.87E-07
**ASV_35_Prevotellaceae**	0.00120586	0.00014444	0.00049642	0.00258541
**ASV_55_Prevotellaceae**	0.00176403	7.98E-05	0.00016266	9.88E-06
**ASV_26_Rhodopirillaceae**	0.00207911	4.33E-05	0.00019498	0.00374657
**ASV_16_Ardenticatenaceae**	0.0031209	7.12E-05	0.00018291	0.0041166
**ASV_14_JG30-KF-CM45**	0.00355299	0.00028447	0.00068267	0.00569495
**ASV_19_Moraxellaceae**	0.00481105	0.00050561	0.00047393	0.00241448
**ASV_4_Moraxellaceae**	0.03536895	0.0020498	0.00677936	0.00261981
**ASV_9_Enterobacteriaceae**	0.00383957	0.00513549	0.00080015	0.00145657
**ASV_11_Iamiaceae**	0.00283444	0.00029221	0.00113889	0.00706611
**ASV_15_Intrasporangiaceae**	0.00342575	0.00029785	0.00052165	0.00410253
**ASV_41_Saccharimonadaceae**	0.00074253	6.61E-05	0.00041051	0.00225363
**ASV_18_Peptostreptococcaceae**	0.00296703	0.00019994	0.00103732	0.00344741
**ASV_23_Family_XIII**	0.00176832	0.00020247	0.00113603	0.00378163
**ASV_2_Mycoplasmataceae**	0.32241279	0.16666138	0.23599444	0.5009397
**ASV_33_Lactobacillaceae**	0.00241586	0.0004051	0.00101525	0.00044841
**ASV_17_Lactobacillaceae**	0.00473274	0.00093646	0.0012226	0.00027108
**ASV_37_Lactobacillaceae**	0.00212166	0.00040242	0.00071918	0.00041614
**ASV_47_Acidaminococcaceae**	0.0005336	6.36E-05	0.00027901	0.0023868
**ASV_51_Lachnospiraceae**	0.00091127	3.57E-05	0.00044789	0.00170769
**ASV_60_Lachnospiraceae**	0.00201715	0.00030984	0.0003198	7.46E-06
**ASV_10_Lachnospiraceae**	0.0031756	0.0008901	0.0029829	0.00704077
