# Supplementary material for: Longitudinal assessment of the bovine ocular bacterial community dynamics in calves
Source: Anim Microbiome. 2021 Jan 30;3:16. doi: 10.1186/s42523-021-00079-3 (PMC7847012; doi:10.1186/s42523-021-00079-3)
Supplement: Supplementary file 12 — Additional file 12: Figure S12. Distribution of the calves age throughout the duration of the trial. [file 42523_2021_79_MOESM12_ESM.pdf]

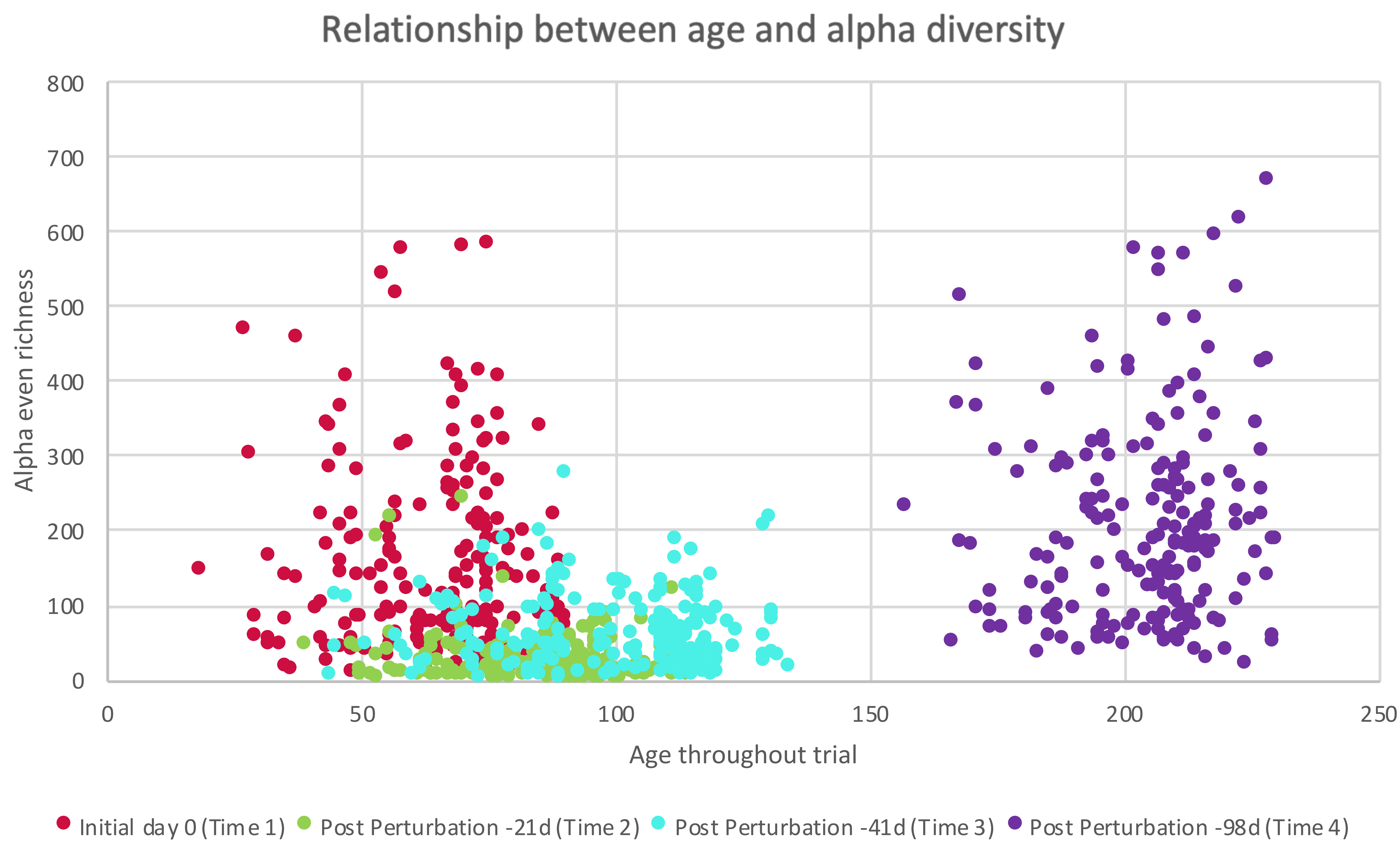
Relationship between age and alpha diversity
800
700
600
500
400
300
200
100
0
Alpha even richness
0
50
100
150
200
250
Age throughout trial
Initial day 0 (Time 1)
Post Perturbation -21d (Time 2)
Post Perturbation -41d (Time 3)
Post Perturbation -98d (Time 4)
