# Supplementary material for: Longitudinal assessment of the bovine ocular bacterial community dynamics in calves
Source: Anim Microbiome. 2021 Jan 30;3:16. doi: 10.1186/s42523-021-00079-3 (PMC7847012; doi:10.1186/s42523-021-00079-3)
Supplement: Supplementary file 13 — Additional file 13: Table S1. Scores of ulcers from cattle infected with IBK during the study. [file 42523_2021_79_MOESM13_ESM.pdf]

Animal_ID	Eye_Infected	Ulcer_Score
8026F	RT	6.0
8031F	LT	1.0
8033F	RT	6.0
8041F	LT	5.0
8042F	LT	5.0
8046F	RT	5.0
8078F	LT	2.0
8080F	LT	0.5
8085F	LT	1.5
8111F	LT	3.0
8123F	LT	5.0
8146F	RT	3.5
8153F	RT	6.0
8181F	LT	3.5
8198F	RT	6.0
8199F	RT	5.0
8202F	LT	3.5
8206F	LT	1.0
8215F	RT	3.5
